# Spatial analysis of individual- and village-level sociodemographic characteristics associated with age at marriage among married adolescents in rural Niger

**DOI:** 10.1186/s12889-020-08759-6

**Published:** 2020-05-19

**Authors:** Holly B. Shakya, John Weeks, Sneha Challa, Paul J. Fleming, Beniamino Cislaghi, Lotus McDougal, Sabrina C. Boyce, Anita Raj, Jay G. Silverman

**Affiliations:** 1 Center on Gender Equity and Health, University of California San Diego, 9100 Gilman Dr, La Jolla, CA USA; 2 San Diego State University, 5500 Campanile Dr, San Diego, CA 92182 USA; 3 University of Michigan, 500 S State St, Ann Arbor, MI 48109 USA; 4 London School of Hygiene and Tropical Medicine, Keppel St, Bloomsbury, London, WC1E 7HT UK; 5 University of California Berkeley, 50 University Ave Hall #7360, Berkeley, CA 94720 USA

**Keywords:** Social norms, Early marriage, Niger, Hot spot analysis, Spatial analysis, geographically weighted regression

## Abstract

**Background:**

Niger has the highest prevalence of child marriage in the world. While child marriage in Niger is clearly normative in the sense that it is commonly practiced, the social and contextual factors that contribute to it are still unclear.

**Methods:**

Here, we tested the importance of village-level factors as predictors of young age at marriage for a group of married adolescent girls (*N* = 1031) in the Dosso district of rural Niger, using multi-level and geographic analyses. We aggregated significant individual level factors to determine whether, independent of a girl’s own sociodemographic characteristics, the impact of each factor is associated at the village level. Finally, we tested for spatial dependence and heterogeneity in examining whether the village-level associations we find with age at marriage differ geographically.

**Results:**

The mean age of marriage for girls in our study was 14.20 years (SD 1.8). Our statistical results are consistent with other literature suggesting that education is associated with delayed marriage, even among adolescent girls. Younger ages at marriage are also associated with a greater age difference between spouses and with a greater likelihood of women being engaged in agricultural work. Consistent with results at the individual level, at the village level we found that the proportion of girls who do agricultural work and the mean age difference between spouses were both predictive of a lower age at marriage for individual girls. Finally, mapping age at marriage at the village level revealed that there is geographical variation in age at marriage, with a cluster of hot spots in the Hausa-dominated eastern area where age at marriage is particularly low and a cluster of cold spots in the Zarma-dominated western areas where age at marriage is relatively high.

**Conclusions:**

Our findings suggest that large-scale approaches to eliminating child marriage in these communities may be less successful if they do not take into consideration geographically and socially determined contextual factors at the village level.

## Background

Niger has the highest prevalence of child marriage in the world; according to the 2012 Demographic and Health Survey 79% of women aged 20–24 are married by the age of 18, with 27% of these marriages having occurred before the girl reached 15 years of age [1]. These child brides quickly become child mothers: 69% of women aged 20–24 years give birth as adolescents (i.e., before reaching age 20). As a result, Niger has the highest adolescent fertility rate in the world with 186 births per 1000 adolescents aged 15–19 [2]. Indeed, Niger is in a pocket of high adolescent fertility. Its next-door neighbors Mali (to the west) at 169 and Chad (to the east) at 161 have the second and third highest levels of adolescent fertility in the world. While child marriage in Niger and neighboring countries is clearly normative in the sense that it is commonly practiced, the social and contextual factors that contribute to it are still unclear.

Childbearing initiated in adolescence continues into adulthood. Niger has the highest total fertility rate in the world, with an average of 6.95 children born to each woman as of 2015–2020, a rate which has remained largely unchanged for the last 70 years [3, 4]. Unsurprisingly in this context of high fertility, contraceptive use is extremely low: only 12% of married women report modern contraceptive use [1, 5]. Less than 100 years ago, life expectancy at birth for females in Niger and other proximal countries was no higher than 20 years [6]. At this level of mortality, half of all children ever born would be dead by age 5, and many of the mothers would have died in childbirth. Early marriage and high fertility were obvious ways to ensure that enough children were born to at least maintain the population size [7]. Life expectancy in Niger and within many of its neighbors is still lower than elsewhere in the world, but it is now in the 50s, well above the level at which such high fertility is necessary for group survival. Yet, these high fertility patterns persist, helping to maintain Niger’s position as one of the poorest countries in the world [8] and one of the fastest growing in population size, as well [9].

Research documents substantial linkages between child marriage, early childbearing, and adverse health consequences for both adolescent mothers and their children [10]. Specifically, girls who marry early have increased risk for sexually transmitted diseases, cervical cancer, obstetric fistulas, death during childbirth, and their children are at risk for premature death [11]. Compounding these vulnerabilities, the high levels of child marriage and fertility in Niger exist in a context of an extremely understaffed health system. The density of doctors, nurses and midwives serving its population of 24 million is among the lowest in the world [12, 13]. These factors all contribute to some of the most sobering health statistics of any country. Niger ranks 17th highest globally in terms of maternal mortality ratio, at 553 maternal deaths per 100,000 live births, and has the 14th highest under-five mortality rate, at 85 deaths per 1000 live births [14, 15].

Consistent with most contexts in which child marriage and high fertility are the norm, gender equity is extremely compromised in Niger. According to the UN, Niger ranks as one of the top three most gender inequitable countries in the world [16]. While education and literacy are low overall, they are substantially lower among women. Only 9% of women have attained at least secondary education (compared with 20% of men) and only 14% of women are literate (compared with 42% of men) [1].

Lower levels of education are associated with child marriage both within Niger and across the region, although because much of the impetus for child marriage is normative, countries in which girls’ education has become a priority have still been unable to reduce rates of child marriage [1, 17–20]. A similar relationship is seen with household wealth. Over the last twenty-five years, West Africa has seen a decrease in child marriage among the wealthiest 20% of women, while in the poorest 20%, levels of child marriage have actually increased over time [21]. Residence is also strongly associated with child marriage in West Africa, where child marriage tends to be concentrated in rural, rather than urban, areas [18, 21]. Child marriage also varies by ethnic group. For example, high prevalence of child marriage in Niger is seen among the Hausa (the country’s largest ethnic group), while lower prevalence is seen amongst the Tuareg (the country’s third largest ethnic group) [18]. These geographic and economic factors influence decisions on marriage via the tradition of a “bride price” offered by grooms’ families, thus creating direct economic incentive to girls’ families for early marriage. In addition to the indirect benefits of one less family member to feed and care for, there is strong motivation to eliminate the risk of social stigma associated with premarital sex or pregnancy [22, 23]. The majority of first marriages in Niger are arranged by parents [24, 25]. While social norms are an important driver of child marriage, they are also reinforced through its practice. Girls who are married early may be more likely to endorse unequal gender norms as a result [20]. Finally, migration experiences likely influence the risk of child marriage, since as younger generations migrate away from their natal communities toward urban areas in search of employment, they lessen their exposure to traditional norms regarding social rules and expectations [26].

It is critical to recognize however, that child marriage and fertility rates are not uniform throughout Niger. Median age at marriage ranges from 15.4 to 19.5 across Niger’s eight first-level administrative regions, and similar variations are seen in other key health and equity measures [1, 18]. Not only are child marriage and fertility influenced by myriad social and structural factors, these factors, and their relationships with one another, vary across social and geographic contexts suggesting social normative influences that transcend simple sociodemographic associations [27–30]. Spatial demographers assume that place is an important determinant of attitudes and behaviors, both because geographic features can inhibit or facilitate behaviors (for instance distance to a health clinic) and perhaps more importantly, because it is through spatial clustering of people that clustering of norms typically occurs [26] [31]. While people with similar characteristics typically choose to interact with each other, a concept known as homophily [32], people who are geographically proximal to each other can also become more alike due to shared exposures or direct social influence. From a spatial analytic perspective, this is captured by the concept of spatial dependence: People in close proximity are more likely to share certain characteristics in common with each other than with people who live at a greater distance [33]. Spatial heterogeneity (also known as spatially varying relationships) on the other hand, refers to situations in which relationships among variables differ according to place [33]. The existence of spatial dependence and/or spatial heterogeneity can be important markers for patterns of social norms.

In norms theory, we understand individual behavior to be determined by the attitudes, expectations, and behaviors of important others, or those within a person’s reference group [34]. Ideally, in norms research, reference groups would be identified through the use of discrete social network ties [35, 36], but in much health and development research such data are lacking. Instead, researchers looking for evidence of norms often generate data with measures across cruder social units in which social ties are inferred. For example, they may examine residents of the same village or neighborhood (the concept behind DHS clusters) to determine whether there is inter-cluster variation. As a proxy for when discrete social network ties are not available, high levels of variation across these spatial units can be viewed as evidence of variability in norms [37]. This is also true of spatial heterogeneity. If, for example, the impact of education on fertility levels varies by geographic area, this suggests that there may be geography-specific social effects that are driving the behavior beyond the expected association of educational levels and fertility [38]. These sorts of insights have important implications for policy applications, as varying levels of social reinforcement and expectations around behaviors of interest can suggest very different strategies for engaging with behavioral change.

The goal of this paper is to use data from a unique source to identify village-level factors that may be associated with age at marriage for a group of already married adolescent girls in rural Niger, controlling for individual-level characteristics. In other words, we want to test the importance of the village social context as a factor influencing child marriage. Understanding these potential normative influences across geographic areas within a country is a critical next step in dissecting coverage of essential health services and determinants of health outcomes, while also allowing for a more nuanced and informed means of addressing gender and health inequities [39]. We specifically ask: controlling for individual level factors, what village level factors associated with age at marriage in rural Niger?

## Methods

### Data collection and sampling

**Fig. 1:**
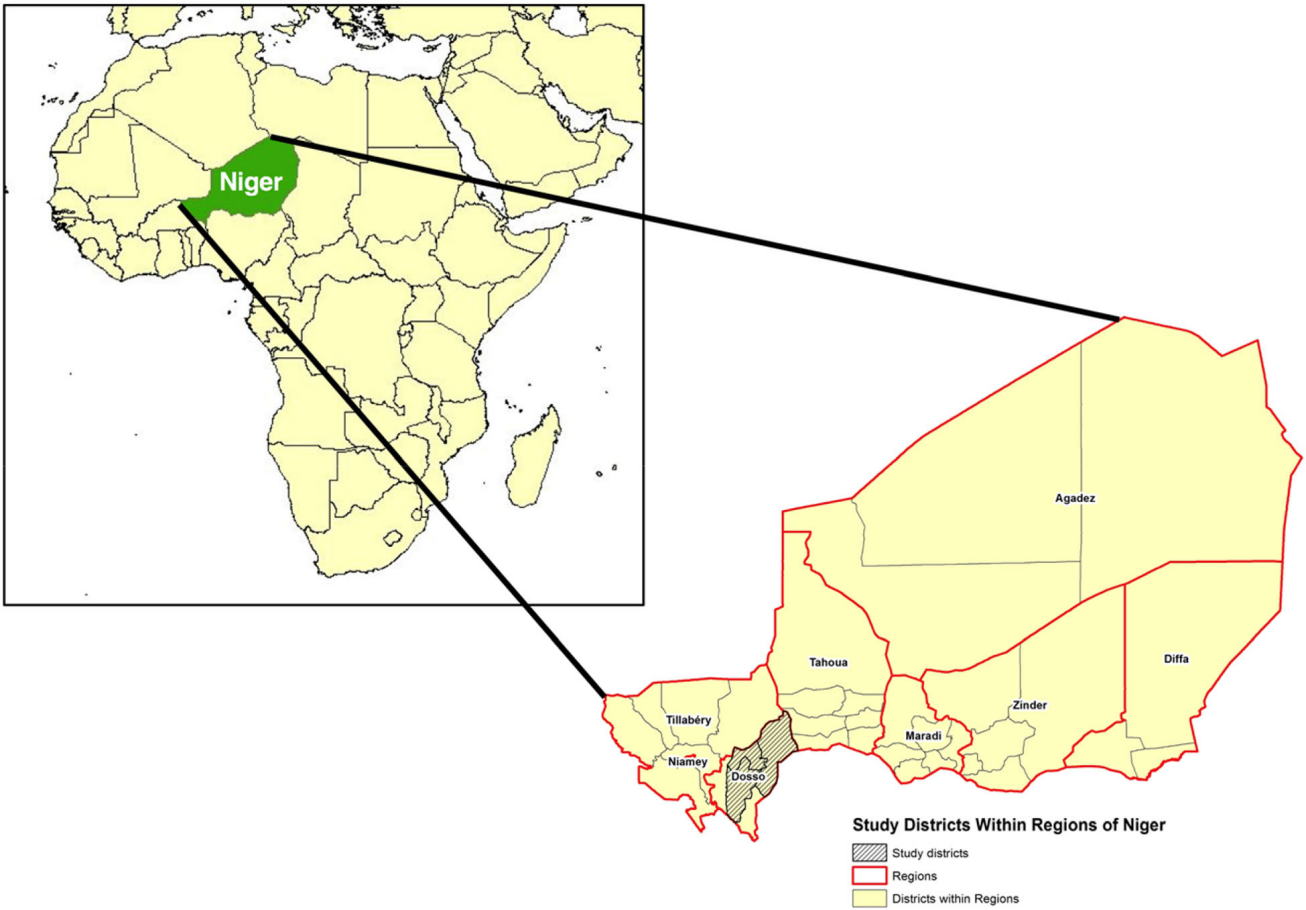
Map showing the three study districts within the Dosso region of Niger, in West Africa

In this research, we analyze baseline data (pre-intervention implementation) that were collected in 2016 from respondents in 48 villages in the Dosso, Doutchi, and Loga districts in the Dosso region of Niger as a cluster randomized control trial (RCT - ClinicalTrials.gov NCT03226730) evaluating a family planning promotion intervention to be implemented by Pathfinder International. From each of the three districts, 16 villages were randomly selected based on the following inclusion criteria: 1) having at least 1000 permanent inhabitants; 2) primarily Hausa or Zarma-speaking (the two major languages of Niger); and 3) no known implementation of interventions focused on family planning or female empowerment. Both intervention and control villages from the RCT are included in this analysis. The study area is shown in Fig. 1.

The sample comprised adolescent wife (aged 13–19 years)-husband dyads (*N* = 1031). A list of married female adolescents was provided by the chief of each village and participants were selected using a random number generator. Eligibility criteria for the married female adolescents included: 1) aged 13–19 years old; 2) married; 3) fluent in Hausa or Zarma; 4) residing in the village where recruitment was taking place, with no plans to move away in the next 18 months nor plans to travel for more than 6 months during that period; 5) not currently sterilized; and 6) providing informed consent to participate in the study. Household-level information was collected from each head of household (Additional File 1) then, *gender-matched trained research assistants from the Dosso region fluent in French and Hausa and/or Zarma conducted surveys separately with the young women (*Additional File 2*) and their husbands.* Of those who were randomly selected, 88.0% participated in the baseline survey. There were no significant differences found in wives’ ages, husbands’ ages, or migration between those who did and did not participate. An equal number of respondents was chosen from each of the three districts. Details on data collection procedures can be found in the published manuscript describing the protocol of this study [40].

### Empirical model and methods

#### Statistical approach

We first ran multivariate linear regression analyses looking at the sociodemographic predictors of age at marriage at the individual level. For all individual measures, we tested between-village variation, using a − 2 log likelihood ratio test in which we compared the − 2 log likelihood of a null model against a multilevel model clustering on the village. For those variables which showed significant between-village variation, we created village-level aggregate measures. To assess which village-level factors to keep in our models, we first ran bivariate analyses testing each village-level factor with individual age at marriage. For those that were significant at *p* < 0.10, we ran the analyses again with the full set of individual sociodemographic factors. We then ran multilevel models looking at the association between village- and individual-level factors and individual age at marriage, clustering on the village. To assess the proportion of variance explained at the individual and village levels we calculated intra-class correlation coefficients for each model. We also calculated the variance inflation factor (VIF) to test whether the individual models were compromised by multi-collinearity.

For those village-level factors that were significantly associated with individual-level age at marriage, we then tested their significance with village-level mean age at marriage to determine which factors would be appropriate in a spatial model. We used hot spot analysis in ArcGIS to assess whether there were significant spatial hotspots of younger age at marriage. Finally, we ran geographically weighted regression to determine whether the factors that were statistically significantly associated with mean age at marriage at the village level varied geographically. All maps in this publication were created by the authors using a licensed version of ArcGIS software 10.7.1.

### Measures

#### Dependent variable

The dependent variable in the analysis is the age at which the wife had married her husband, a continuous variable.

#### Individual-level sociodemographic predictors

Sociodemographic data were collected from each head of household, most often the husband, but in some cases, this included both the husband’s and wife’s reports. As a large age difference between a husband and wife is often associated with gender inequality, we included the difference between husbands’ and wives’ ages in years. We measured modern education of both husbands and wives as a numeric measure from 0 to 3, with 0 representing no formal schooling, 1-incomplete primary, 2-completed primary, and 3 as past primary. We also included a binary measure of Quranic education for both husbands and wives. We assessed family wealth using the standard household assets list [41], summing each item that was reported in the home: a watch, a mobile phone, a bicycle, a motorbike or scooter, a car or truck, or an animal drawn cart. We asked wives if they had worked outside of the home in the past 12 months. Work reported was almost exclusively agricultural. We also included a measure of food insecurity that asked whether in the last month the respondent or any member of the respondent’s family went without eating the whole day because there was not enough food. As a measure of agency, we included a question for women asking who chose her spouse for her, categorizing it as she herself solely chose or did not. Finally, we included the number of children born to that couple, whether or not the couple lived with the extended family, the number of wives the husband had, and ethnic group. These variables are summarized below in Table 1.

**Table 1:** Descriptive statistics *N* = 1031

	Mean	SD	%
Wife’s current age	17.32	1.54	
Husband’s current age	25.54	5.39	
Wife’s education 0–3	0.51	0.79	
Husband’s education 0–3	0.74	0.89	
Wife decides spouse			29%
Quranic school Wives			25%
Quranic school Husbands			34%
Wife’s age at marriage	14.20	1.82	
Wife’s age at first birth	15.72	1.93	
Age difference between spouses	8.22	5.01	
Household assets 0–6	2.05	1.16	
Food insecurity			22%
Wife agricultural labor			41%
Number of children ever born	0.91	0.98	
Live with extended family			82%
Polygamous			13%
Tribe Hausa			30%
Tribe Zarma			70%
District Dosso			32%
District Doutchi			33%
District Loga			35%

#### Village-level aggregate measures

For each of the individual sociodemographic variables, we created non-self, village-level aggregate measures, either means or proportions depending upon the variables, including the mean of the dependent variable age at marriage. Non-self means and proportions are those in which the village-level aggregate value for each individual is calculated minus their own value and divided by N-1.

## Descriptive characteristics

Excluding participants with missing data, our final sample included 1031 couples. Table 1 shows the descriptive characteristics of the sample population. The mean age of wives within the sample population was 17.32 years (SD 1.54), while the mean age of husbands was 25.54 (SD 5.34). Husbands thus were on average 8.22 years (SD 5.01) older than their wives. The mean age at marriage for women was 14.20 years of age (SD 1.82) and 13% of marriages were polygamous. The mean age at first birth was 15.72 (SD 1.93), and at the time of the interview 60.4% of these adolescent wives had given birth to at least one child.

## Results

### Sociodemographic associations with wife’s age at marriage

We ran a multivariate linear regression model to determine the main sociodemographic predictors of a wife’s age at marriage (see Table 2).^1^ Consistent with other research [1, 17–20], women’s education was inversely related to age at marriage. Women who reported agricultural work were likely to have married almost 1 year younger (− 0.85 years, 95% CI -0.63 - -1.07) than those who did not report agricultural work, while women who were in polygamous marriages were likely to have married almost 1 year older (1.10 years, 95% CI 0.75–1.46) compared to those still in monogamous marriages. Also, of note were the significant district-level effects: those in Doutchi were likely to marry at a younger age (0.86 years, 95% CI 0.19–1.54) than those in Loga or Dosso. This difference is related to the fact that Hausa marry younger than Zarma, and almost all Hausa couples in the sample lived in Doutchi, whereas almost all Zarma couples lived in either Dosso or Loga. We found no significant associations with age at marriage and husband’s education, household asset status, or household food security. Using the variance inflation factor test in R, we determined that the only variables for which there was collinearity were being of Hausa ethnicity and living in Doutchi. Therefore, we dropped the District variable from the analyses.

**Table 2:** Demographic associations with age at marriage: multivariate linear regression individual level analysis. N = 1031

	Coef	SE	P
Age difference husband-wife	−1.22	0.15	0.00
Age difference husband-wife quadratic	0.01	0.00	0.00
Wife choose husband	−0.20	0.12	0.09
Education Wife	0.17	0.05	0.00
Wife agricultural work	−0.86	0.11	0.00
Polygamous	1.15	0.18	0.00
Education Husband	−0.03	0.06	0.58
Household assets	0.10	0.06	0.08
Food security	0.03	0.13	0.79
Extended family	0.15	0.14	0.29
Qur’anic education Husband	−0.14	0.13	0.26
Qur’anic education Wife	0.10	0.14	0.47
Tribe Hausa (ref)			
Tribe Zarma	0.39	0.13	0.00

Dependent variable = wife’s age at marriage

### Between-village variation

The − 2 log likelihood test for between-village variation was significant for every factor clustered on village: wife’s age at marriage as well as individual sociodemographic characteristics. We ran bivariate analyses for each village-level factor and individual age at marriage. Then we ran analyses that included those variables that were significant at *p* < 0.10 in separate models including the full set of individual-level factors (see Table 3). The proportion of girls who reported choosing their spouse, the proportion of girls who reported agricultural work, mean age difference between spouses, and mean household assets were the factors we ultimately included in our final models. Table 4 shows the village-level means and proportions of these variables as well as their ranges. Mean age at marriage in the population sample as a whole was 14.21 years, while the mean age at marriage across villages ranged from 12.42–15.93. Similarly, while the average difference in age between spouses was 8.18 years, the mean across villages ranged from 4.82–12.09 years. The proportion of women who reported agricultural work varied the most between villages, with some villages having no women who reported agricultural work, while in others all women reported agricultural work.

**Table 3:** Results from separate multilevel analyses testing village level predictors with individual age at marriage

	Coef	SE	P
Proportion who do agricultural work	−0.44	0.10	0.00
Mean age difference between spouses	−0.20	0.10	0.06
Proportion of wives choosing spouse	−0.19	0.10	0.06
Mean household assets	0.20	0.09	0.04
Proportion report food insecurity	0.06	0.10	0.52
Proportion with Qur’anic education men	−0.06	0.12	0.61
Proportion with Qur’anic education women	0.09	0.14	0.52

**Table 4:** Village level means and proportions of characteristics associated with marriage at a younger age, 48 villages

	Mean	Range
Age at first marriage	14.21	12.42–15.93
Age difference between spouses	8.18	4.82–12.09
Women agricultural work	0.42	0.00–1.00
Wife decides spouse	0.28	.13–0.82
Household assets	1.96	1.12–3.20

### Village-level aggregate measures as predictors of age at marriage

In Table 5 we show the results of our full multilevel model analysis of village- and individual-level factors as predictors of age at marriage. Model 1 shows the null model, with an intraclass-correlation coefficient (ICC) of 16%, suggesting that 16% of the variance in an individual girl’s age at marriage is accounted for by factors at the village level. In Model 2 we include the four village-level variables that we constructed from the significant individual predictors of age at marriage. We find that the proportion of polygamous marriages in the village and mean level of women’s education lose significance when included in a model with mean age difference between spouses and village-level proportion of wives who do agricultural work. In this model, 7.5% of the unexplained variation in age at marriage still occurs at the village level, even after inclusion of several village-level factors. In Model 3, we included only individual-level factors. The ICC was 9%, suggesting that some of the variance in age at marriage at the village level can be explained by village-level differences in those individual factors. Finally, Model 4 includes village-level and individual-level factors. Inclusion of the proportion of women who work in the village and the village-level mean age difference between spouses in the model with individual characteristics accounts for 33% of the unexplained village level variance from Model 3 (a reduction from 9 to 6%). The individual-level characteristics identified in the earlier model retain significance while additionally we understand that marriage age differs by village, associated partially with the proportion of women in that village who do agricultural labor and the village-level average difference in age between spouses. A one standard deviation increase in the proportion of the village women who do agricultural work is associated with a 0.42 year decrease in marriage age. This could be interpreted as evidence that there is a demand for labor in strongly agricultural villages that is being met by bringing in young women as wives of the males in the household, keeping in mind that 82% of the respondents live in extended households. Because husband-level factors are subsequent to the marriage, we reran the individual level analyses (see the online appendix), removing husband level factors and including age at first menarche, since a young age at menarche has been associated with a higher likelihood of child marriage and adolescent pregnancy [42], in the analyses. The results are consistent with those of our initial analyses.

**Table 5:** Multi level model showing individual and village level characteristics predictive of age at marriage, and proportion of variance accounted for

	Null Model (1)	Village level only (2)			Individual characteristics (3)			Individual and Village characteristics (4)		
		Coef	SE	P	Coef	SE	P	Coef	SE	P
Household assets village level mean		0.02	0.10	0.84						
Wife decides spouse village level proportion		−0.02	0.11	0.83						
Proportion of women agricultural work village		−0.40	0.10	0.00				−0.38	0.10	0.00
Mean age difference between spouses village		−0.32	0.09	0.00				−0.13	0.09	0.16
Age difference husband/wife					−1.17	0.15	0.00	−1.15	0.15	0.00
Age difference husband/wife quadratic					0.01	0.00	0.00	0.01	0.00	0.00
Wife decides spouse					−0.14	0.12	0.26	−0.05	0.12	0.66
Quranic education Husband					−0.09	0.12	0.47	−0.09	0.12	0.45
Quranic education Wife					0.02	0.14	0.89	0.02	0.14	0.90
Education Wife					0.21	0.07	0.00	0.20	0.07	0.00
Education Husband					−0.02	0.06	0.69	−0.01	0.06	0.90
Household assets					0.05	0.05	0.29	0.03	0.05	0.51
Food security					0.05	0.13	0.70	0.02	0.13	0.87
Wife agricultural work					−0.57	0.12	0.00	−0.36	0.14	0.01
Extended family					0.16	0.13	0.23	0.17	0.13	0.22
Number of wives					0.92	0.16	0.00	0.94	0.16	0.00
Tribe Hausa (ref)										
Tribe Zarma					0.37	0.20-	0.07	0.39	0.21	0.06
Group Level Variance	0.55	0.23			0.30			0.19		
Individual Level Variance	2.81	2.81			2.54			2.53		
ICC	16%	7.5%			11%			7%		
AIC	4075	4052			3978			3964		

### Spatial analysis

**Fig. 2:**
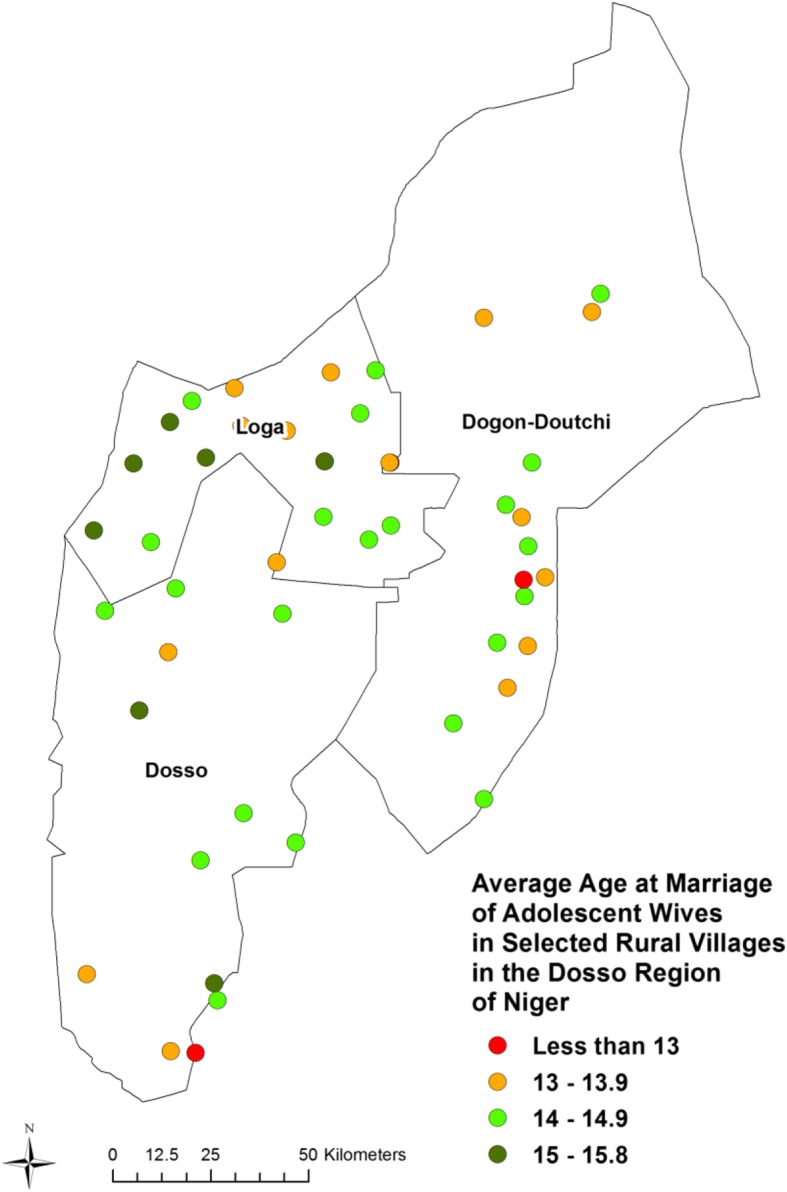
Mean age at marriage from within a population of married adolescent girls within selected villages in Southwest Niger. Note that mean age at marriage varies significantly by village, although geographically those differences seem somewhat random with possible clustering of higher age at marriage in the Northwest, and younger age at marriage in the Northeast

**Fig. 3:**
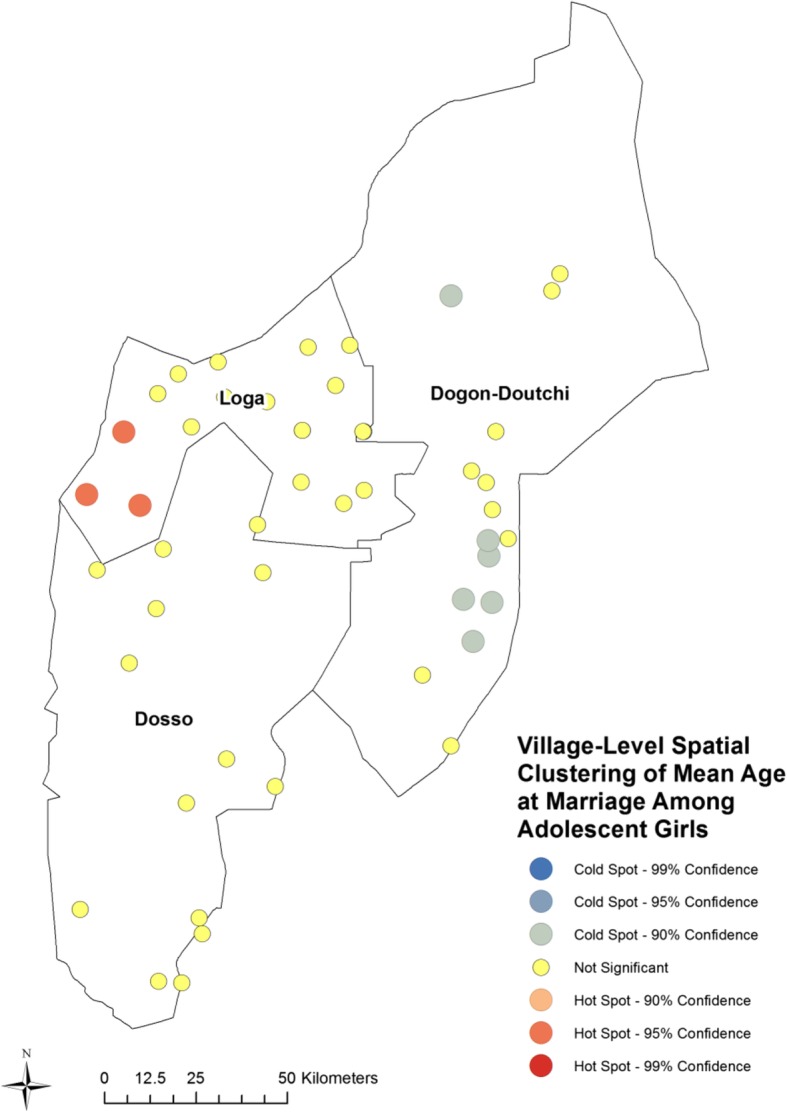
Geographic clustering of mean age at marriage among married adolescent girls in Southwest Niger. Clustering determined using Getis-Ord Gi Hot Spot Analysis in ArcMap with a fixed distance band determination for clustering. Note clusters of very young age at marriage near the eastern border with Nigeria in the Hausa-dominated Doutchi district. These results confirm a small cluster of lower age at marriage in the East, and a small cluster of higher age at marriage in the West, in the Zarma-dominated district of Loga

Figure 2 is a map of the study population, depicting the geographic difference in village-level mean age at marriage. Note that mean age at marriage varies significantly by village, although geographically those differences seem somewhat random with possible clustering of higher age at marriage in the western part of the region, especially in Loga, and younger age at marriage in the eastern side of the region. To determine whether there is significant spatial clustering of mean age at marriage across villages, we conducted a hot spot analysis using Getis-Ord Gi Hot Spot Analysis in ArcMap with a fixed distance band determination for clustering. Our results confirm the existence of a cluster of villages in the western edge of the district in which the age at marriage is higher (i.e., a hot spot), and a cluster of villages with low age at marriage (i.e., a cold spot) in the eastern part of the region (see Fig. 3). The clusters of very young age at marriage are near the eastern border with Nigeria in the Hausa-dominated Doutchi district. The adjacent area of northwestern Nigeria is the region of that country with the highest fertility and lowest age at marriage.

**Fig. 4:**
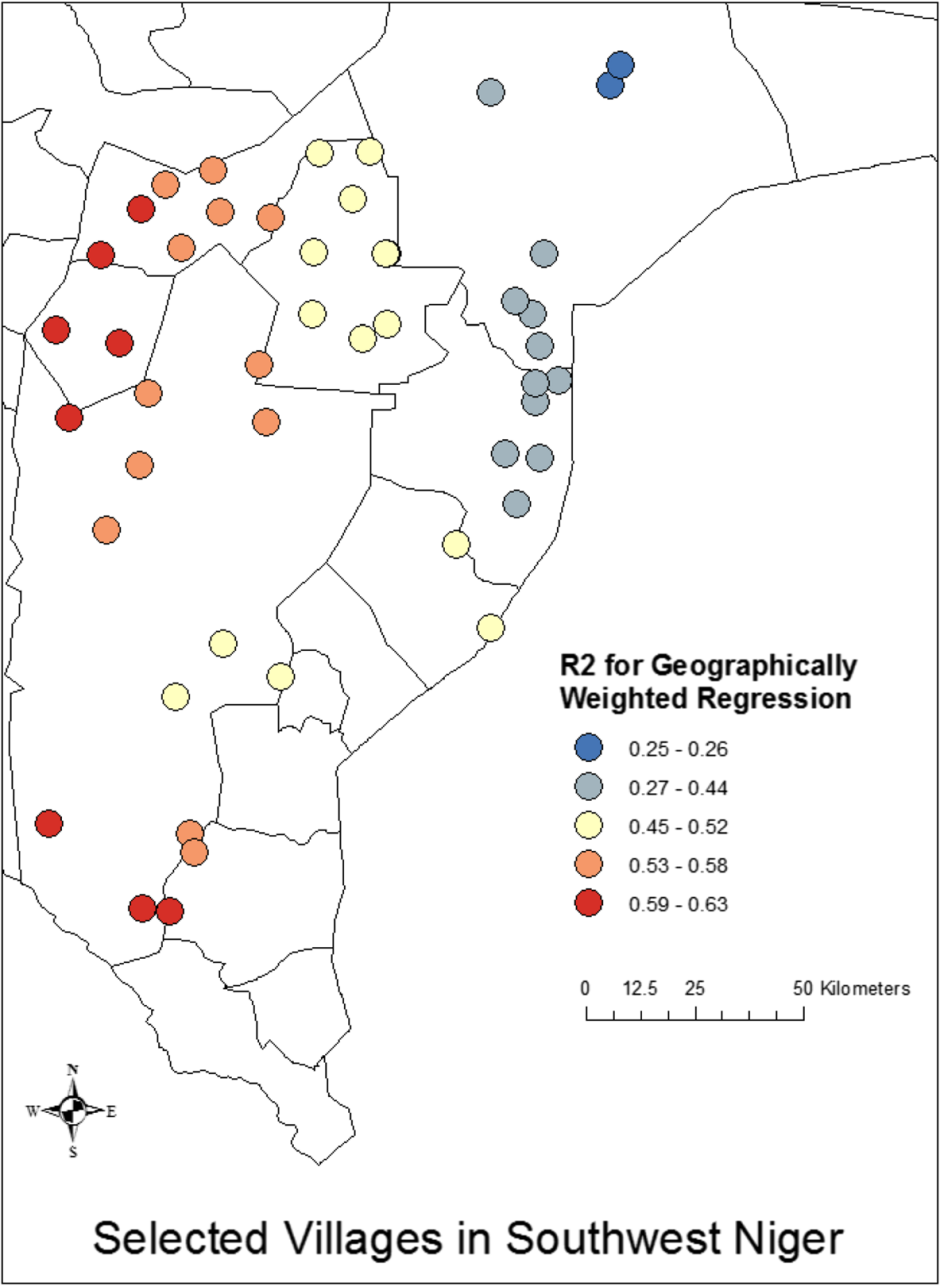
Using the results of the linear model (Table 5) we ran a geographically weighted regression to assess spatial trends in the associations with mean age at marriage at the village level using proportion of girls who do agricultural work and the mean age difference between spouses as the primary predictors. Note that the full model explains a greater proportion of the variance in mean age at marriage in the Zarma-dominated areas in the west compared to the Hausa-dominated areas in the east

**Fig. 5:**
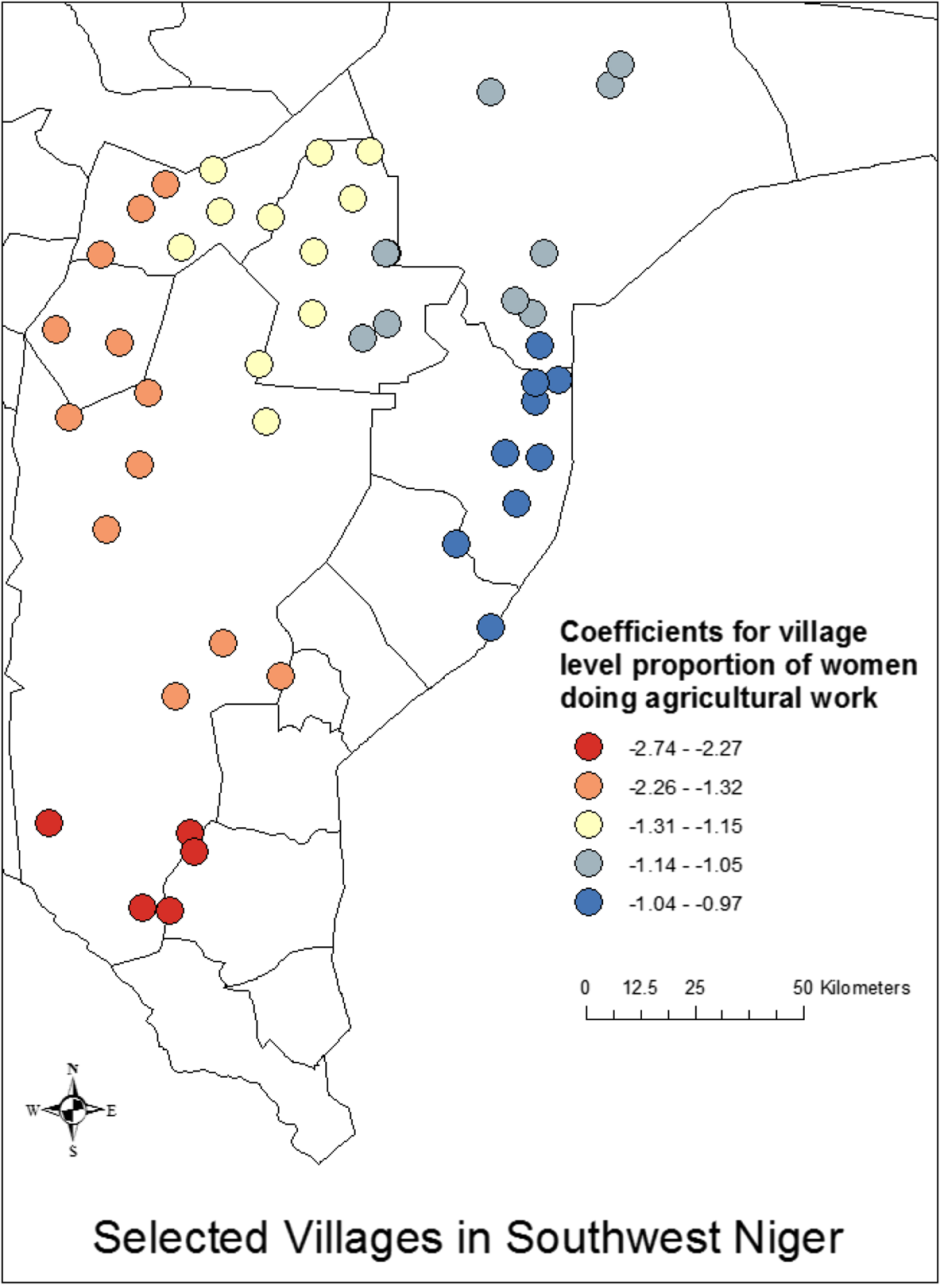
Relationship between proportion of women in a village doing agricultural work and mean age at marriage at the village level. Overall, proportion of women doing agricultural work is associated with a lower mean age at marriage, with the strongest effects being in the South and the West

**Fig. 6:**
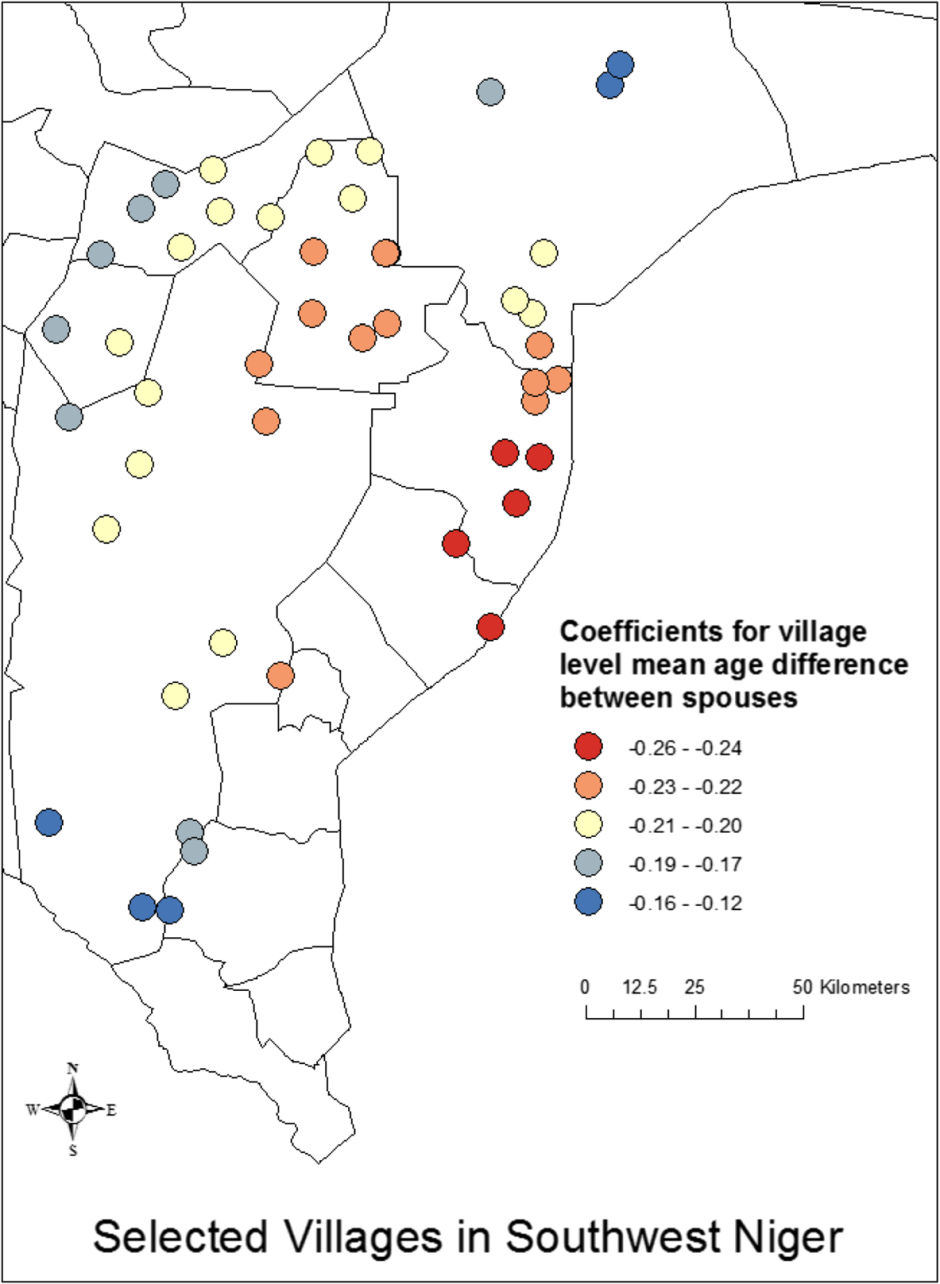
Village level mean age difference between spouses is a more strongly associated with younger mean age at marriage in the East compared to the West, although across geographic areas it is a significant risk factor

We then ran a geographically weighted regression analysis, building upon the results of the OLS model shown in Table 6 in which we identify the mean village age difference between spouses and the proportion of women who do agricultural work as significant predictors of village-level mean age at marriage (R^2^ = 0.52). Our analysis suggests that the village-level factors do vary geographically in their influence on age at marriage, with the stronger associations taking place in the western regions and weaker associations taking place in the eastern regions. We find that the R^2^ for the analysis in the western regions is greater by a factor of more than 2 compared to the R^2^ for the same analysis in the eastern regions (0.59–0.63 vs 0.25–0.26), suggesting that there are factors in the eastern region that are associated with age at marriage that are not accounted for in the ordinary linear models (Fig. 4). When we break down this analysis by individual independent variables (Figs. 5 and 6), we see that the coefficients for both village-level age difference between spouses and the village proportion who engage in agricultural work vary geographically, with the patterns apparent in the proportion who engage in agricultural work being more consistent with the overall model.

**Table 6:** Village level linear regression showing the association between village level aggregate factors and village level mean age at marriage. N = 48

Village Level Predictor of Village Level age at Marriage	R^2^	?	p
Mean village age difference	−0.38	0.12	0.00
Proportion of girls who do agricultural work	− 0.50	0.12	0.00
Proportion of village girls decide spouse	−0.03	0.14	0.84
Mean household assets	0.01	0.12	0.93

## Discussion

The purpose of this paper has been to examine the factors associated with marriage age patterns of currently married Nigerien adolescent girls living in the Dosso region. We use a unique dataset from an area with one of the highest rates of child marriage in the world to analyse the individual-level, village-level, and spatial factors associated with age at marriage to gain an understanding of how and where girls are more likely to marry at younger or older ages. Our results highlight significant regional differences with important implications for efforts to reduce child marriage in Niger.

We find that girls with more education and currently in polygamous marriages are likely to marry older, while those with greater age difference between themselves and their husbands, who live in the Doutchi district of Dosso, and who report engagement in agricultural work are more likely to marry younger. At the village level, however, we found that only two of our tested covariates were significant. The proportion of girls who do agricultural work and the mean age difference between spouses were both predictive of a lower age at marriage for a girl. Finally, mapping age at marriage at the village level revealed that there is some geographical variation in age at marriage for married adolescents in the Dosso region, with a cluster of cold spots in the Hausa-dominated eastern area where age at marriage is particularly low and a cluster of hot spots in the Zarma-dominated western areas where age at marriage is relatively high. We also found geographic differences in the strength of the association between the village-level factors predictive of younger age at marriage. The Dosso region of Niger, with a population of 2,368,651 in 2016, comprises primarily agricultural communities producing millet, and these communities are known to experience high rates of poverty [43].

Each of the three districts of the Dosso region in which our study has been conducted – Dogondoutchi (Doutchi), Dosso, and Loga [44] has a slightly different ethnic breakdown with Doutchi being primarily Hausa and Dosso and Loga primarily Zarma. These cultural differences may help to explain the village-level differences our work shows. Villages with lower ages at marriage are concentrated in the Doutchi district, where economic hardship has created the social conditions conducive to maintaining the historical pattern of younger ages of marriage. This area of Niger is economically unstable because the soil is not very fertile, and there is limited access to water sources. The decreased outputs of treenut cultivation in comparison to millet farming could explain the clustering of villages with lower age at marriage in Doutchi, as families with fewer economic resources often need to marry their daughters off earlier to alleviate financial strains [18]. The results of our geographically weighted regression suggest that this district of the Dosso region is also the one in which a greater mean age difference between spouses at the village level is most strongly associated with younger age at marriage for girls. These contextual stressors, which in and of themselves compromise maternal and child health and survival, are likely to further compound and compromise maternal and child health through mechanisms of heightened risk associated with earlier (vs. later) adolescent childbearing [45, 46].

Large age differences between spouses have been associated with inequality in spousal relations in many contexts [47–50]. The larger the age difference between spouses, the less empowered a girl is to advocate for herself, the higher the likelihood that she is exploited for labor in the husband’s household, and the more vulnerable she is to violence. We see in our analysis that not only is the age difference associated with younger age at marriage at the individual level for girls, it is predictive at the village level as well. In other words, we see evidence that there are social contexts in which some of the variation in age at marriage is associated with contextual-level variation of age difference between spouses. To be sure, we do not have data about the household characteristics of the young wives’ parental homes, but it is likely that they are marrying into households that are similar to the one in which they were raised.

There was a clear geographic difference in the association between village-level factors and village-level mean age at marriage. The model was most strongly predictive in the western area of the region, and most weakly predictive in the eastern area of the region, with an estimated R^2^ less than half of that in the western area. When we look at the coefficients for the two separate predictors, we see that geographic variation in the coefficients for proportion of village doing agricultural work almost directly matches the variation in the R^2^, while the variation in mean age difference between spouses is almost entirely reversed, having a stronger association in the eastern area, and a weaker association in the western area. This would suggest that much of the power for the geographic dispersion of the model is coming from the proportion of the village doing agricultural work. It is also interesting to note that the model is least predictive where average age at marriage is youngest. So, what explains this variation? Where average age at marriage is younger, these other village-level factors may be less consequential. Furthermore, the geographic dispersion of statistical associations between village-level factors and village-level mean age at marriage seems more pronounced than the hot spots of marriage age. Clearly there is clustering of village-level factors influencing these associations which have not been captured in our data. Spatial heterogeneity of this sort is indicative of norms, although we do not have the evidence to definitively say that is the case. Villages can be a crude proxy for reference groups, and when we see village-level factors that are significantly predictive, it is necessary to look carefully for norms. We have, through our extensive modeling of village-level factors, ruled out many reasonable possibilities such as average level of education or household assets. These factors are essentially subsumed under the factor of agricultural work or not. Unmeasured geographic factors that impact agricultural work may combine with normative expectations around marriageability, reinforced by geographically contingent reference groups. Future research will be necessary to understand these differences.

While girls’ education was significantly predictive of older age at marriage at the individual level, it was not significant at the village level. This may be simply because girls who marry later have more opportunity for education. It is also possible that because our sample consisted of married adolescents, the range of educational achievement that could be captured in our sample and then aggregated at the village level was limited and not dispersed enough to uncover this sort of higher-level association. Only 4% of our sample went on to anything past primary school, which may also be a result of the general lack of educational resources in these rural areas. Nevertheless, while education is associated with older age at marriage, because much of what drives child marriage is normative, simple investments in girls’ education has not been sufficient in many contexts to drive notable decreases in child marriage [19]. *Our results suggest that this may also be the case in rural Niger.* Longitudinal analysis will be necessary in order to understand the dynamic relationship between education and age at marriage within these contexts.

### Limitations

While our study spanned a large number of villages (*N* = 48), these villages were chosen from a limited geographic area in southwestern Niger. A broader geographic dispersion would be helpful for gaining a better understanding of these contextual issues. While these results may not be generalizable outside of this specific context, the high rates of poverty and low levels of education are typical of many contexts in which child marriage is common, and therefore our results can be taken as evidence to inform similar studies in similar contexts. Our sample consisted of girls who were already married so we cannot identify the factors that distinguish those who marry as adolescents from those who do not. Despite this limitation, it is nonetheless the case that because such a high proportion of girls in Niger marry as adolescents (approximately 3 in 4), an understanding of the factors associated with the timing of adolescent marriage can be an important contribution to this field. Finally, because our data are not longitudinal, we cannot assess time-dependent changes that would be more indicative of causality. Despite this limitation, our analysis provides important evidence for possible causal mechanisms that can potentially be useful to policy-planners and health providers.

## Conclusion

While all of the girls in this sample were married as adolescents, age at marriage varied significantly among them, and this variation was significant at the village level. Mean age at marriage was clustered by village in a way that suggests important contextual differences in this outcome. Our results show that some of this difference is associated with sociocultural differences we were able to measure such as the mean age difference between spouses and the prevalence of agricultural work in the community, along with the cultural factor of ethnicity (Zarma or Hausa). Our geographic analysis points to the clustering of other characteristics at the village level that may have important impacts on age at marriage and the degree to which these factors vary geographically in that impact. The results suggest that large-scale approaches to eliminating child marriage in these communities may be less successful if they do not take into consideration geographically and socially determined contextual factors at the village level. Current recommendations for child marriage interventions are largely community-based but do not target village-level norms [51–54]. These efforts may be inadequate to maintain change in the absence of efforts to target norms in the social context beyond the family. Considerations of village-level clustering and spatial distribution of child marriage norms is crucial for better understanding and then addressing this issue.

## Supplementary information

**Additional file 1.** Original household survey used for data collection.

**Additional file 2.** Original survey for data collection from female participants.

## Data Availability

The datasets generated and/or analysed during the current study are not publicly available due to concerns regarding privacy and the sensitive nature of the data but are available from the corresponding author on reasonable request.

## Abbreviations

CI: Confidence interval
SD: Standard deviation
ICC: Intraclass correlation
IRB: Institutional review board

## Footnotes

1 In our initial exploration of the data, we found evidence of a non-linear relationship between spousal age difference and age at marriage, which led us to include a quadratic term in the model. We found that as the age difference between spouses increases, the age at marriage for an individual wife tends to decrease. However, when the age difference reaches approximately 15 years of age difference (about 10% of the population) this trend reverses, and individual age at marriage slightly increases.
