# Supplementary material for: Spatial analysis of individual- and village-level sociodemographic characteristics associated with age at marriage among married adolescents in rural Niger
Source: BMC Public Health. 2020 May 19;20:729. doi: 10.1186/s12889-020-08759-6 (PMC7238637; doi:10.1186/s12889-020-08759-6)
Supplement: Supplementary file 1 — Additional file 1. Original household survey used for data collection. [file 12889_2020_8759_MOESM1_ESM.docx]

| **FEMALE SURVEY** | | | |
| --- | --- | --- | --- |
| **B. HOUSEHOLD DECISION MAKING** | | | |
| 1. B_YRSMAR |  | How long ago did you marry your husband? | [Number] Years |
| 1. B_WHOHUSB |  | Who had the greatest say with regard to arranging your marriage to your husband? | 1 RESPONDENT CHOSE |
|  |  |  | 2 RESPONDENT & HUSBAND CHOSE EACH OTHER |
|  |  |  | 3 RESPONDENT WITH SOMEONE ELSE CHOSE |
|  |  |  | 4 RESPONDENT'S FAMILY CHOSE |
|  |  |  | 5 HUSBAND OR HIS FAMILY CHOSE RESPONDENT |
|  |  |  | 6 SOMEONE ELSE CHOSE |
|  |  |  | 7 JOINT DECISION NOT INCLUDING RESPONDENT |
|  |  |  | 997 OTHER |
|  |  |  | 998 DON'T KNOW |
|  |  |  | 999 DECLINE TO ANSWER |
| 1. B_ASKHUSB | If _WHOHUSB=4-7, 997, 998. or 999 | When your current husband was being chosen for you were you asked whether you wanted to marry him or not? | 1 YES |
|  |  |  | 2 NO |
|  |  |  | 997 OTHER |
|  |  |  | 999 DECLINE TO ANSWER |
| 1. B_DECISMONEY |  | Who usually decides how your husband's earnings will be used? | 1 RESPONDENT |
|  |  |  | 2 HUSBAND |
|  |  |  | 3 RESPONDENT & HUSBAND JOINTLY |
|  |  |  | 4 RESPONDENT'S MOTHER-IN-LAW |
|  |  |  | 5 RESPONDENT'S FATHER-IN-LAW |
|  |  |  | 6 CO-WIFE |
|  |  |  | 7 JOINT DECISION NOT INCLUDING RESPONDENT |
|  |  |  | 997 OTHER |
|  |  |  | 998 DON'T KNOW |
|  |  |  | 999 DECLINE TO ANSWER |
| 1. B_DECISHLTH |  | Who usually makes decisions about health care for yourself (for example, whether you can receive treatment at the CS or CSI)? | 1 RESPONDENT |
|  |  |  | 2 HUSBAND |
|  |  |  | 3 RESPONDENT & HUSBAND JOINTLY |
|  |  |  | 4 RESPONDENT'S MOTHER-IN-LAW |
|  |  |  | 5 RESPONDENT'S FATHER-IN-LAW |
|  |  |  | 6 CO-WIFE |
|  |  |  | 7 JOINT DECISION NOT INCLUDING RESPONDENT |
|  |  |  | 997 OTHER |
|  |  |  | 998 DON'T KNOW |
|  |  |  | 999 DECLINE TO ANSWER |
| 1. B_DECISPURCH |  | Who usually makes decisions about making major household purchases (for example, buying livestock)? | 1 RESPONDENT |
|  |  |  | 2 HUSBAND |
|  |  |  | 3 RESPONDENT & HUSBAND JOINTLY |
|  |  |  | 4 RESPONDENT'S MOTHER-IN-LAW |
|  |  |  | 5 RESPONDENT'S FATHER-IN-LAW |
|  |  |  | 6 CO-WIFE |
|  |  |  | 7 JOINT DECISION NOT INCLUDING RESPONDENT |
|  |  |  | 997 OTHER |
|  |  |  | 998 DON'T KNOW |
|  |  |  | 999 DECLINE TO ANSWER |
| 1. B_DECISVISIT |  | If you should visit your family or relatives, who makes that decision? | 1 RESPONDENT |
|  |  |  | 2 HUSBAND |
|  |  |  | 3 RESPONDENT & HUSBAND JOINTLY |
|  |  |  | 4 RESPONDENT'S MOTHER-IN-LAW |
|  |  |  | 5 RESPONDENT'S FATHER-IN-LAW |
|  |  |  | 6 CO-WIFE |
|  |  |  | 7 JOINT DECISION NOT INCLUDING RESPONDENT |
|  |  |  | 997 OTHER |
|  |  |  | 998 DON'T KNOW |
|  |  |  | 999 DECLINE TO ANSWER |
| 1. B_HUSBHELP |  | During the last week, has your husband helped you with any of the household responsibilities (for example, cleaning, cooking, caring for children)? | 1 YES |
|  |  |  | 2 NO |
|  |  |  | 997 OTHER |
|  |  |  | 998 DON'T KNOW |
|  |  |  | 999 DECLINE TO ANSWER |
| 1. B_HSHTASKA |  | How often did you do the following tasks for your family in the past 1 month: Washing clothes | 1 Never |
|  |  |  | 2 1-2 times |
|  |  |  | 3 2-15 times |
|  |  |  | 4 Daily |
|  |  |  | 998 DON'T REMEMBER |
|  |  |  | 999 DECLINE TO ANSWER |
| 1. B_HSHTASKB |  | How often did you do the following tasks for your family in the past 1 month: Cleaning the house | 1 Never |
|  |  |  | 2 1-2 times |
|  |  |  | 3 2-15 times |
|  |  |  | Daily |
|  |  |  | 998 DON'T REMEMBER |
|  |  |  | 999 DECLINE TO ANSWER |
| 1. B_HSHTASKC |  | How often did you do the following tasks for your family in the past 1 month: Preparing food | 1 Never |
|  |  |  | 2 1-2 times |
|  |  |  | 3 2-15 times |
|  |  |  | Daily |
|  |  |  | 998 DON'T REMEMBER |
|  |  |  | 999 DECLINE TO ANSWER |
| 1. B_CHILDCARA | if = birthtot>=1 | Who typically performs the daily care of the child? Please indicate if it was USUALLY YOU, if it was SHARED EQUALLY with your spouse, or it was USUALLY YOUR SPOUSE who performed this task. | 1 USUALLY ME |
|  |  |  | 2 SHARED EQUALLY |
|  |  |  | 3 USUALLY SPOUSE |
|  |  |  | 997 OTHER |
|  |  |  | 998 DON'T REMEMBER |
|  |  |  | 999 DECLINE TO ANSWER |
| **C. FINANCIAL DECISION MAKING** | | |  |
| 1. C_DECISEARN |  | If you should work to gain money, who authorizes in the family? | 1 RESPONDENT |
|  |  |  | 2 HUSBAND |
|  |  |  | 3 RESPONDENT & HUSBAND JOINTLY |
|  |  |  | 4 RESPONDENT'S MOTHER-IN-LAW |
|  |  |  | 5 RESPONDENT'S FATHER-IN-LAW |
|  |  |  | 6 CO-WIFE |
|  |  |  | 7 JOINT DECISION NOT INCLUDING RESPONDENT |
|  |  |  | 997 OTHER |
|  |  |  | 998 DON'T KNOW |
|  |  |  | 999 DECLINE TO ANSWER |
| 1. C_OWNLAND |  | Please tell me if you alone, or jointly with your husband own land or the house you live in | 1 DOES NOT OWN |
|  |  |  | 2 OWNS JOINTLY |
|  |  |  | 3 OWNS ALONE |
|  |  |  | 997 OTHER |
|  |  |  | 999 DECLINE TO ANSWER |
| 1. C_OWNANML |  | Please tell me if you alone, or jointly with your husband own livestock such as a goat or camel | 1 DOES NOT OWN |
|  |  |  | 2 OWNS JOINTLY |
|  |  |  | 3 OWNS ALONE |
|  |  |  | 997 OTHER |
|  |  |  | 999 DECLINE TO ANSWER |
| 1. C_WSELL | IF _OWNLAND=2,3 OR If _OWNANML=2,3 | IF OWNS OR JOINTLY OWNS, If you ever need to, can you sell this asset/these assets without anyone else’s permission? | 1 YES |
|  |  |  | 2 NO |
|  |  |  | 997 OTHER |
|  |  |  | 998 DON'T KNOW |
|  |  |  | 999 DECLINE TO ANSWER |
| 1. C_WMONEY |  | Do you have any money of your own that you alone can decide how to use? | 1 YES |
|  |  |  | 2 NO |
|  |  |  | 997 OTHER |
|  |  |  | 999 DECLINE TO ANSWER |
| 1. C_HUNGRYAM |  | In the last month, did you or any members of your family go without eating the whole day because there was not enough food? | 1 YES |
|  |  |  | 2 NO |
|  |  |  | 998 DON'T KNOW |
|  |  |  | 999 DECLINE TO ANSWER |
| 1. C_MOBMKT |  | Are you usually permitted to go to the following places on your own, only if someone accompanies you, or not at all? To the local market? | 1 ALONE |
|  |  |  | 2 ONLY ACCOMPANIED |
|  |  |  | 3 NOT AT ALL |
|  |  |  | 997 OTHER |
|  |  |  | 999 DECLINE TO ANSWER |
| 1. C_MOBCSI |  | Are you usually permitted to go to the following places on your own, only if someone accompanies you, or not at all? To the casa sante or CSI? | 1 ALONE |
|  |  |  | 2 ONLY ACCOMPANIED |
|  |  |  | 3 NOT AT ALL |
|  |  |  | 997 OTHER |
|  |  |  | 999 DECLINE TO ANSWER |
| 1. C_MOBVISIT |  | Are you usually permitted to go to the following places on your own, only if someone accompanies you, or not at all? To homes of friends in the village? | 1 ALONE |
|  |  |  | 2 ONLY ACCOMPANIED |
|  |  |  | 3 NOT AT ALL |
|  |  |  | 997 OTHER |
|  |  |  | 999 DECLINE TO ANSWER |
| **D. FERTILITY INFORMATION** | |  |  |
| 1. D_MENARC |  | Have you had your first menstrual period? | 1 YES |
|  |  |  | 2 NO |
|  |  |  | 998 DON'T KNOW |
|  |  |  | 999 DECLINE TO ANSWER |
| 1. D_MENARCTIME | if _MENARC=1 | (IF YES) How long ago did you have your first menstrual period? | Number |
| 1. D_BRTHANY |  | Now I would like to ask about all the births you have had during your life. Have you ever given birth? | 1 YES |
|  |  |  | 2 NO |
|  |  |  | 999 DECLINE TO ANSWER |
| 1. D_BIRTHTOTAL | If _BRTHANY=1 | In total, how many births have you had in your life, including to children who may not still be alive? | Number |
| 1. D_BRTHYRS |  | Tell me about your birth [#]. How long ago was your birth [#]? | Number |
| 1. D_PREGTIMING |  | Was this baby born at the same time as another baby (for example, twins, triplets)? | 1 YES |
|  |  |  | 2 NO |
|  |  |  | 999 DECLINE TO ANSWER |
| 1. _NOT A QUESTION | if _TWIN=1 | For the purposes of these next questions we will talk about one of those children and then I'll ask you the same questions about the other(s). | NOT A QUESTION |
| 1. D_BRTHSEX |  | Was this baby a boy or a girl? | 1 BOY |
|  |  |  | 2 GIRL |
|  |  |  | 999 DECLINE TO ANSWER |
| 1. D_BRTHDEATH |  | Sometimes women give birth to a baby who is born alive but then dies later (for example, a baby who cries or shows signs of life but does not survive). Is this child [#] still alive? | 1 YES |
|  |  |  | 2 NO |
|  |  |  | 999 DECLINE TO ANSWER |
| 1. D_BRTHDEATHB |  | How old was this child when (he/she) died? | Number |
| 1. D_BRTHNME | if _BRTHDEATH=1, 999 | (IF YES) What name was given to your baby [#]? | Text |
| 1. D_BRTHRCT | If _LIVSON>0 OR _LIVDAU>0 | Have you had any births since the last birth you just told me about? | 1 YES |
|  |  |  | 2 NO |
|  |  |  | 999 DECLINE TO ANSWER |
| 1. D_MISCAR |  | Have you ever had a pregnancy that miscarried or ended in a stillbirth? | 1 YES |
|  |  |  | 2 NO |
|  |  |  | 999 DECLINE TO ANSWER |
| 1. D_MISCARNUM |  | How many pregnancies have you had that miscarried or ended in a stillbirth? | Number |
| 1. D_MENSTA | if total in _BIRTHTOTAL>=1 | Has your menstrual period returned since the birth of (NAME OF LAST BIRTH)? | 1 YES |
|  |  |  | 2 NO |
|  |  |  | 998 DON'T KNOW |
|  |  |  | 999 DECLINE TO ANSWER |
| 1. D_MENSTB | if total in _BIRTHTOTAL>1 | (IF MORE THAN ONE BIRTH) Did your period return between the birth of (NAME OF SECOND TO LAST BIRTH) and (NAME OF LAST BIRTH)? | 1 YES |
|  |  |  | 2 NO |
|  |  |  | 998 DON'T KNOW |
|  |  |  | 999 DECLINE TO ANSWER |
| 1. D_MENSTC | if total in _BIRTHTOTAL>=1 | For how many months after the birth of (NAME OF LAST BIRTH) did you not have a period? | Number |
| 1. D_SEX_NO | if total in _BIRTHTOTAL>=1 | For how many months after the birth of (NAME OF LAST BIRTH) did you not have sexual intercourse? | Number |
| 1. D_MENST_LAST | if _MENARC=1 | How long ago did your last menstrual period start? | Number |
| **E. PREGNANCY DESIRE** |  |  |  |
| 1. E_BRTHWANT | If _BRTHANY=1 | When you got pregnant the last time, was the timing as you hoped? Did you wish to get pregnant at that time, wish to wait longer since your last pregnancy before getting pregnant again, or did you wish to not have any more pregnancies. | 1 AT THAT TIME |
|  |  |  | 2 WAIT LONGER |
|  |  |  | 3 NO MORE PREGNANCIES |
|  |  |  | 997 OTHER |
|  |  |  | 999 DECLINE TO ANSWER |
| 1. E_BRTHWAIT | if _BRTHWANT=2 | (IF WAIT LONGER) How much longer did you want to wait? | Number |
| 1. E_NUMCHILD |  | If you could choose the exact number of children to have in your whole life, regardless of how many children you have currently, how many would that be? | Number |
| 1. E_MORCHA | if _PREG=2, 998, 999 | IF NOT CURRENTLY PREGNANT, Would you like to have (a/another) child, or would you prefer not to have any (more) children? | 1 HAVE (ANOTHER) CHILD |
|  |  |  | 2 NO MORE/NONE |
|  |  |  | 3 SAYS SHE CAN'T GET PREGNANT |
|  |  |  | 997 OTHER |
|  |  |  | 998 UNDECIDED |
|  |  |  | 999 DECLINE TO ANSWER |
| 1. E_MORCHB | if _PREG=1 | IF CURRENTLY PREGNANT After the child you are expecting now, would you like to have another child, or would you prefer not to have any more children? | 1 HAVE ANOTHER CHILD |
|  |  |  | 2 NO MORE/NONE |
|  |  |  | 3 SAYS SHE CAN'T GET PREGNANT |
|  |  |  | 997 OTHER |
|  |  |  | 998 UNDECIDED |
| 1. E_MORCHTIM | If _MORCHA=1 OR _MORCHB=1 | IF YES How long would you like to wait from now until you become pregnant? | 1 None (want to get pregnant now) |
|  |  |  | 2 Less than a year |
|  |  |  | 3 1-2 years |
|  |  |  | 4 2-5 years |
|  |  |  | 5 More than 5 years |
|  |  |  | 997 OTHER |
|  |  |  | 998 UNDECIDED |
|  |  |  | 999 DECLINE TO ANSWER |
|  |  |  |  |
| 1. F_TYPE |  | Did you know that there are things that women can do to space or delay becoming pregnant? | 1 YES |
|  |  |  | 2 No, did not know |
|  |  |  | 997 OTHER |
|  |  |  | 999 DECLINE TO ANSWER |
| 1. F_CHW12 |  | In the last 12 months, has a community health worker visited you and talked to you about your health? | 1 YES |
|  |  |  | 2 NO |
|  |  |  | 999 DECLINE TO ANSWER |
| 1. F_CHW12_2 | IF _CHW12=1 | IF YES How many times? | 1 Yes, received 1 visit |
|  |  |  | 2 Yes, received 2-5 visits |
|  |  |  | 3 Yes, received more than 5 visits |
|  |  |  | 997 OTHER |
|  |  |  | 998 DON'T KNOW |
|  |  |  | 999 DECLINE TO ANSWER |
| 1. F_CHW3 | IF _CHW12=1 | In the last 3 months, has a community health worker visited you and talked to you about your health? | 1 YES |
|  |  |  | 2 NO |
|  |  |  | 999 DECLINE TO ANSWER |
| 1. F_CHW3_2 | IF _CHW3=1 | IF YES How many times? | 1 Yes, received 1 visit |
|  |  |  | 2 Yes, received 2-3 visits |
|  |  |  | 3 Yes, received more than 3 visits |
|  |  |  | 997 OTHER |
|  |  |  | 998 DON'T KNOW |
|  |  |  | 999 DECLINE TO ANSWER |
| 1. F_CHWTOPIC | IF _CHW12=1 | IF CHW VISITED When the community health worker came, what topics were discussed? | 1 Topics related to FAMILY PLANNING |
|  |  |  | 2 Topics related to NUTRITION |
|  |  |  | 3 Topics related to GENDER (examples, female empowerment, men's responsibility to assist their wives, roles of men and women in the household, etc.) |
|  |  |  | 4 Topics related to PREGNANCY HEALTH |
|  |  |  | 5 The pregnancy I had at that time |
|  |  |  | 997 OTHER |
|  |  |  | 998 DON'T KNOW |
|  |  |  | 999 DECLINE TO ANSWER |
| 1. F_CHWFP | IF _CHW12=1 | IF CHW VISITED Did the community health worker provide you with any form of family planning? | 1 YES |
|  |  |  | 2 NO |
|  |  |  | 999 DECLINE TO ANSWER |
| 1. F_CHWFP2 | IF _CHWFP=1 | IF YES What type? | 1 PILL |
|  |  |  | 2 CONDOM |
|  |  |  | 3 FEMALE CONDOM |
|  |  |  | 997 OTHER |
|  |  |  | 998 DON'T KNOW |
|  |  |  | 999 DECLINE TO ANSWER |
| 1. F_CHWACC | IF _CHW12=1 | IF CHW VISITED Did the community health worker accompany you to the CS/CSI to help you get a form of family planning? | 1 YES |
|  |  |  | 2 NO |
|  |  |  | 999 DECLINE TO ANSWER |
| 1. F_CHWACC_2 | IF _CHWACC=1 | IF YES What type of family planning did they take you there to get? | 1 IUD |
|  |  |  | 2 INJECTIBLES |
|  |  |  | 3 IMPLANTS |
|  |  |  | 4 PILL |
|  |  |  | 5 CONDOM |
|  |  |  | 6 FEMALE CONDOM |
|  |  |  | 7 EMERGENCY CONTRACEPTION |
|  |  |  | 997 OTHER |
|  |  |  | 998 DON'T KNOW |
|  |  |  | 999 DECLINE TO ANSWER |
| 1. F_CHWHELP | IF _CHW12=1 | (IF YES TO RECEIVED VISIT FROM CHW) Did you find the visit from a community health worker helpful? | 1 YES |
|  |  |  | 2 NO |
|  |  |  | 998 Not Sure |
|  |  |  | 999 DECLINE TO ANSWER |
| 1. F_CHWYES | If _CHWHELP=1, 998 | IF YES What was helpful? | 1 The information was useful to help me make choices about my life |
|  |  |  | 2 The information helped me to speak to my husband about family planning |
|  |  |  | 3 The information helped to better understand how to keep me and my family healthy |
|  |  |  | 4 The information taught me how to healthily space my pregnancies |
|  |  |  | 997 OTHER |
|  |  |  | 998 DON'T KNOW |
|  |  |  | 999 DECLINE TO ANSWER |
| 1. F_CHWYESOTH | If _CHWYES=997 | Other_______ | Text |
| 1. F_CHWNO | _CHWHELP=2 | (IF NO) Why not? | 1 I AM NOT INTERESTED IN FAMILY PLANNING |
|  |  |  | 2 I ALREADY KNEW THE INFORMATION SHE TOLD ME |
|  |  |  | 3 THE VISIT CAUSED PROBLEMS FOR ME |
|  |  |  | 997 OTHER |
|  |  |  | 998 DON'T KNOW |
|  |  |  | 999 DECLINE TO ANSWER |
| 1. F_CHWNOOTH | if _CHWNO=997 | Other_______ | Text |
| 1. F_CHWDIS | IF _CHW12=1 | (IF YES TO RECEIVED VISIT FROM CHW) Was anyone in the household, for example your husband, co-wives, in-laws, unhappy or disapproving that the health worker came to speak to you? | 1 YES |
|  |  |  | 2 NO |
|  |  |  | 998 DON'T KNOW |
|  |  |  | 999 DECLINE TO ANSWER |
| 1. F_CHWDISWHO | if _CHWDIS=1 | (IF YES) Who was unhappy or disapproving? | 1 HUSBAND |
|  |  |  | 2 YOUR PARENTS |
|  |  |  | 3 MOTHER IN LAW |
|  |  |  | 4 FATHER IN LAW |
|  |  |  | 5 CO-WIFE |
|  |  |  | 997 OTHER |
|  |  |  | 998 DON'T KNOW |
|  |  |  | 999 DECLINE TO ANSWER |
| 1. F_CHWWANT | IF _CHW12=2 | (IF NO TO RECEIVED VISIT FROM CHW) Would you ever want a health worker to come and talk to you about healthy timing and spacing of pregnancy or family planning? | 1 YES |
|  |  |  | 2 NO |
|  |  |  | 998 DON'T KNOW |
|  |  |  | 999 DECLINE TO ANSWER |
| 1. F_CHWWDIS | IF _CHW12=2 | (IF NO TO RECEIVED VISIT FROM CHW) Would someone in your family disapprove of a health worker coming to talk to you about healthy timing and spacing of pregnancy or family planning? | 1 YES |
|  |  |  | 1 NO ONE WOULD DISAPPROVE |
|  |  |  | 998 DON'T KNOW |
|  |  |  | 999 DECLINE TO ANSWER |
| 1. F_CHWWDISW | if _CHWWDIS=1 | Who would disapprove of this? | 1 HUSBAND |
|  |  |  | 2 YOUR PARENTS |
|  |  |  | 3 MOTHER IN LAW |
|  |  |  | 4 FATHER IN LAW |
|  |  |  | 5 CO-WIFE |
|  |  |  | 997 OTHER |
|  |  |  | 998 DON'T KNOW |
|  |  |  | 999 DECLINE TO ANSWER |
| 1. F_GRP12 |  | In the last 12 months, have you participated in a discussion group for young women? | 1 YES |
|  |  |  | 2 NO |
|  |  |  | 999 DECLINE TO ANSWER |
| 1. F_GRP12_2 | IF _GRP12=1 | IF YES How many times? | 1 Participated in 1 group |
|  |  |  | 2 Yes, participated in 2-5 groups |
|  |  |  | 3 Yes, participated in more than 5 groups |
|  |  |  | 997 OTHER |
|  |  |  | 998 DON'T KNOW |
|  |  |  | 999 DECLINE TO ANSWER |
| 1. F_GRP3 | IF _GRP12=1 | In the last 3 months, have you participated in a discussion group for young women? | 1 YES |
|  |  |  | 2 NO |
|  |  |  | 999 DECLINE TO ANSWER |
| 1. F_GRP3_2 | IF _GRP3=1 | IF YES How many times? | 1 Participated in 1 group |
|  |  |  | 2 Yes, participated in 2-5 groups |
|  |  |  | 3 Yes, participated in more than 5 groups |
|  |  |  | 997 OTHER |
|  |  |  | 998 DON'T KNOW |
|  |  |  | 999 DECLINE TO ANSWER |
| 1. F_GRPTOPIC | IF _GRP12=1 | During the groups you have attended, what topics were discussed? | 1 Topics related to FAMILY PLANNING |
|  |  |  | 2 Topics related to NUTRITION |
|  |  |  | 3 Topics related to GENDER (examples, female empowerment, men's responsibility to assist their wives, roles of men and women in the household, etc.) |
|  |  |  | 4 Topics related to PREGNANCY HEALTH |
|  |  |  | 997 OTHER |
|  |  |  | 998 DON'T KNOW |
|  |  |  | 999 DECLINE TO ANSWER |
| 1. F_GRPFP | IF _GRP12=1 | Did the group facilitator provide you with any form of family planning? | 1 YES |
|  |  |  | 2 NO |
|  |  |  | 999 DECLINE TO ANSWER |
| 1. F_GRPFP_2 | IF _GRPFP=1 | IF YES What type? | 1 PILL |
|  |  |  | 2 CONDOM |
|  |  |  | 3 FEMALE CONDOM |
|  |  |  | 997 OTHER |
|  |  |  | 998 DON'T KNOW |
|  |  |  | 999 DECLINE TO ANSWER |
| 1. F_GRPHELP | IF _GRP12=1 | (IF YES TO PARTICIPATED IN GROUPS) Did you find the women's discussion groups helpful? | 1 YES |
|  |  |  | 2 NO |
|  |  |  | 998 Not Sure |
|  |  |  | 999 DECLINE TO ANSWER |
| 1. F_GRPYES | If _GRPHELP=1, 998 |  | 1 The information was useful to help me make choices about my life |
|  |  |  | 2 The information helped me to speak to my husband about family planning |
|  |  |  | 3 The information helped to better understand how to keep me and my family healthy |
|  |  |  | 4 The information taught me how to healthily space my pregnancies |
|  |  |  | 997 OTHER |
|  |  |  | 998 DON'T KNOW |
|  |  |  | 999 DECLINE TO ANSWER |
| 1. F_GRPYESOTH | If _GRPYES=997 | Other_______ | Text |
| **G. REPRODUCTIVE HEALTH KNOWLEDGE** | | | |
| 1. G_HLTHYTIM |  | Now I would like to talk about family planning, the ways or methods that a couple can use to space or delay a pregnancy. Please answer to the best of your ability. For the health of the mother and the baby, how much time should a woman wait between giving birth and trying to become pregnant again? | 1 CORRECT (2 years/24 months) |
|  |  |  | 2 INCORRECT |
|  |  |  | 998 DON'T KNOW |
|  |  |  | 999 DECLINE TO ANSWER |
| 1. G_HLTHYMIS |  | For the health of the mother and the baby, how much time should a woman wait between a miscarriage (a pregnancy that did not result in a birth) and trying to become pregnant again? | 1 CORRECT (6 months) |
|  |  |  | 2 INCORRECT |
|  |  |  | 998 DON'T KNOW |
|  |  |  | 999 DECLINE TO ANSWER |
| 1. G_PIL |  | Have you ever heard of the PILL? | 1 YES |
|  |  |  | 2 NO |
|  |  |  | 998 DON'T KNOW |
|  |  |  | 999 DECLINE TO ANSWER |
| 1. G_PILA | if _PIL=1 | I am going to say a statement about this form of family planning that could be true or false. I want you to tell me if you think it is a true statement or if it is a false statement. Birth control pills are effective even if a woman misses taking them for two or three days in a row. | 1 TRUE |
|  |  |  | 2 FALSE |
|  |  |  | 998 DON'T KNOW |
|  |  |  | 999 DECLINE TO ANSWER |
| 1. G_PILB | if _PIL=1 | After a woman stops taking birth control pills, it's possible for her to get pregnant right away. | 1 TRUE |
|  |  |  | 2 FALSE |
|  |  |  | 998 DON'T KNOW |
|  |  |  | 999 DECLINE TO ANSWER |
| 1. G_IUD |  | Have you ever heard of an IUD? | 1 YES |
|  |  |  | 2 NO |
|  |  |  | 998 DON'T KNOW |
|  |  |  | 999 DECLINE TO ANSWER |
| 1. G_IUDA | if _IUD=1 | I am going to say a statement about this form of family planning that could be true or false. I want you to tell me if you think it is a true statement or if it is a false statement. An IUD cannot be felt by a woman’s husband during sex. (True) | 1 TRUE |
|  |  |  | 2 FALSE |
|  |  |  | 998 DON'T KNOW |
|  |  |  | 999 DECLINE TO ANSWER |
| 1. G_IUDB | if _IUD=1 | An IUD can get permanently stuck in a woman’s body. | 1 TRUE |
|  |  |  | 2 FALSE |
|  |  |  | 998 DON'T KNOW |
|  |  |  | 999 DECLINE TO ANSWER |
| 1. G_INJ |  | Have you ever heard of INJECTIBLES or DEPO-PROVERA? | 1 YES |
|  |  |  | 2 NO |
|  |  |  | 998 DON'T KNOW |
|  |  |  | 999 DECLINE TO ANSWER |
| 1. G_INJA | if _INJ=1 | I am going to say a statement about this form of family planning that could be true or false. I want you to tell me if you think it is a true statement or if it is a false statement. Women using the birth control shot, Depo-Provera, must get an injection every three months. | 1 TRUE |
|  |  |  | 2 FALSE |
|  |  |  | 998 DON'T KNOW |
|  |  |  | 999 DECLINE TO ANSWER |
| 1. G_INJB | if _INJ=1 | Using Depo-Provera can cause a woman to never be able to have children again. | 1 TRUE |
|  |  |  | 2 FALSE |
|  |  |  | 998 DON'T KNOW |
|  |  |  | 999 DECLINE TO ANSWER |
| 1. G_IMP |  | Have you ever heard of IMPLANTS? | 1 YES |
|  |  |  | 2 NO |
|  |  |  | 998 DON'T KNOW |
|  |  |  | 999 DECLINE TO ANSWER |
| 1. G_IMPA | if _IMP=1 | I am going to say a statement about this form of family planning that could be true or false. I want you to tell me if you think it is a true statement or if it is a false statement. Long-acting methods like the implant or IUD can be removed early if a woman changes her mind about wanting to get pregnant. | 1 TRUE |
|  |  |  | 2 FALSE |
|  |  |  | 998 DON'T KNOW |
|  |  |  | 999 DECLINE TO ANSWER |
| 1. G_IMPB | if _IMP=1 | If a woman wanted to secretly delay having her next child, an IUD or injectible could be used without anyone in her family knowing. | 1 TRUE |
|  |  |  | 2 FALSE |
|  |  |  | 998 DON'T KNOW |
|  |  |  | 999 DECLINE TO ANSWER |
| 1. G_MCON |  | Have you ever heard of MALE CONDOMS? | 1 YES |
|  |  |  | 2 NO |
|  |  |  | 998 DON'T KNOW |
|  |  |  | 999 DECLINE TO ANSWER |
| 1. G_MCONA | if _MCON=1 | I am going to say a statement about this form of family planning that could be true or false. I want you to tell me if you think it is a true statement or if it is a false statement. It is okay to use the same condom more than once. | 1 TRUE |
|  |  |  | 2 FALSE |
|  |  |  | 998 DON'T KNOW |
|  |  |  | 999 DECLINE TO ANSWER |
| 1. G_LAM |  | Have you ever heard of LACTATIONAL AMENORRHEA METHOD (LAM)? | 1 YES |
|  |  |  | 2 NO |
|  |  |  | 998 DON'T KNOW |
|  |  |  | 999 DECLINE TO ANSWER |
| 1. G_LAMA | if _LAM=1 | I am going to say a statement about this form of family planning that could be true or false. I want you to tell me if you think it is a true statement or if it is a false statement. Breastfeeding is an effective form of delaying pregnancy even after the woman's menstrual bleeding has returned since giving birth | 1 TRUE |
|  |  |  | 2 FALSE |
|  |  |  | 998 DON'T KNOW |
|  |  |  | 999 DECLINE TO ANSWER |
| 1. G_LAMB | if _LAM=1 | Exclusive breastfeeding means the woman breastfeeds her baby “on demand,” day and night, and does not give any other food, water or liquid | 1 TRUE |
|  |  |  | 2 FALSE |
|  |  |  | 998 DON'T KNOW |
|  |  |  | 999 DECLINE TO ANSWER |
| 1. G_FPPLACE |  | Do you know of a place where you can obtain a method of family planning? | 1 YES |
|  |  |  | 2 NO |
|  |  |  | 997 OTHER |
|  |  |  | 999 DECLINE TO ANSWER |
| 1. G_FPWHERE | if _FPPLACE=1 | Where is that? (Check all that apply) | 1 DISTRICT HOSPITAL |
|  |  |  | 2 CENTRE DE SANTÉ/CSI |
|  |  |  | 3 CASE DE SANTÉ/ACS |
|  |  |  | 4 Pharmacie |
|  |  |  | 5 Distribution à Base Communautaire/OTHER COMMUNITY HEALTH WORKER |
|  |  |  | 6 Vendeurs Ambulants |
|  |  |  | 7 Guérisseur traditionnelle |
|  |  |  | 8 FRIEND/RELATIVE |
|  |  |  | 997 OTHER (SPECIFY) |
|  |  |  | 998 DON'T KNOW |
|  |  |  | 999 DECLINE TO ANSWER |
| 1. G_FPWHEREOTH | if_FPWHERE=997 | Other_______ | Text |
| 1. G_FPINFOWHO |  | What person in your life do you trust most when it comes to getting information on spacing or delaying pregnancy? | 1 MOTHER |
|  |  |  | 2 FRIEND |
|  |  |  | 3 SISTER |
|  |  |  | 4 ASC from the CS |
|  |  |  | 5 OTHER COMMUNITY HEALTH WORKER |
|  |  |  | 6 NURSE/DOCTOR AT CSI |
|  |  |  | 7MOTHER IN LAW |
|  |  |  | 8 CO-WIFE |
|  |  |  | 9 HUSBAND |
|  |  |  | 10 VILLAGE LEADER |
|  |  |  | 11 OTHER FEMALE RELATIVE |
|  |  |  | 12 NO ONE |
|  |  |  | 997 OTHER |
|  |  |  | 998 DON'T KNOW |
|  |  |  | 999 DECLINE TO ANSWER |
| 1. G_KWLMENARC |  | I am going to say a statement that could be true or false. I want you to tell me if you think it is a true statement or if it is a false statement. Generally speaking, once a female gets her first menstrual period, she can get pregnant | 1 TRUE |
|  |  |  | 2 FALSE |
|  |  |  | 998 DON'T KNOW |
|  |  |  | 999 DECLINE TO ANSWER |
| 1. G_KWLFERT |  | From one menstrual period to the next, are there certain days when a woman is more likely to become pregnant? | 1 YES |
|  |  |  | 2 NO |
|  |  |  | 998 DON'T KNOW |
|  |  |  | 999 DECLINE TO ANSWER |
| 1. G_KWLFERTA | if _KWLFERT=1 | Is this time just before her period begins, during her period, right after her period has ended, or halfway between two periods? | 1 JUST BEFORE HER PERIOD BEGINS |
|  |  |  | 2 DURING HER PERIOD |
|  |  |  | 3 RIGHT AFTER HER PERIOD HAS ENDED |
|  |  |  | 4 HALFWAY BETWEEN TWO PERIODS |
|  |  |  | 997 OTHER |
|  |  |  | 998 DON'T KNOW |
|  |  |  | 999 DECLINE TO ANSWER |
| 1. G_KWLFERTPOST |  | After the birth of a child, can a woman become pregnant before her menstrual period has returned? | 1 TRUE |
|  |  |  | 2 FALSE |
|  |  |  | 998 DON'T KNOW |
|  |  |  | 999 DECLINE TO ANSWER |
| **H. SEGMENTATION/CONTRACEPTION ACCEPTABILITY** | | |  |
| 1. H_ACCSPAC |  | Do you think that it is okay for a couple to wait 2 years or more in between giving birth to a child and getting pregnant again? | 1 YES |
|  |  |  | 2 NO |
|  |  |  | 998 DON'T KNOW |
|  |  |  | 999 DECLINE TO ANSWER |
| 1. H_ACCWAIT |  | Do you think that it is okay for a young women to wait 2 years or more after getting married to have her first child? | 1 YES |
|  |  |  | 2 NO |
|  |  |  | 998 DON'T KNOW |
|  |  |  | 999 DECLINE TO ANSWER |
| 1. H_ACCLIMIT |  | Do you think that it is okay for a couple to try to limit the number of children they have? | 1 YES |
|  |  |  | 2 NO |
|  |  |  | 998 DON'T KNOW |
|  |  |  | 999 DECLINE TO ANSWER |
| 1. H_FPIMPA |  | Which of the following characteristics of a family planning method would be important for you? It is a natural method. | 1 Important |
|  |  |  | 2 Not important |
|  |  |  | 998 DON'T KNOW |
|  |  |  | 999 DECLINE TO ANSWER |
| 1. H_FPIMPB |  | It is easy to stop at any moment | 1 Important |
|  |  |  | 2 Not important |
|  |  |  | 998 DON'T KNOW |
|  |  |  | 999 DECLINE TO ANSWER |
| 1. H_FPIMPC |  | the method is discrete, no one needs to know that I am using it | 1 Important |
|  |  |  | 2 Not important |
|  |  |  | 998 DON'T KNOW |
|  |  |  | 999 DECLINE TO ANSWER |
| 1. H_FPIMPD |  | the method protects me against sexually transmitted infections | 1 Important |
|  |  |  | 2 Not important |
|  |  |  | 998 DON'T KNOW |
|  |  |  | 999 DECLINE TO ANSWER |
| 1. H_FPCONSID |  | Which of the following methods have you ever considered using? | 1 I have not considered using any method |
|  |  |  | 2 IUD |
|  |  |  | 3 INJECTIBLES |
|  |  |  | 4 IMPLANTS |
|  |  |  | 5 PILL |
|  |  |  | 6 CONDOM |
|  |  |  | 7 FEMALE CONDOM |
|  |  |  | 8 EMERGENCY CONTRACEPTION |
|  |  |  | 9 LACTATION AMENAREA METHOD (LAM) |
|  |  |  | 10 OTHER TRADITIONAL METHOD |
|  |  |  | 11 ABSTINENCE |
|  |  |  | 998 DON'T KNOW |
|  |  |  | 999 DECLINE TO ANSWER |
| **J. BARRIERS TO ACCESSING CONTRACEPTION** | | | |
| 1. J_BARPERM |  | Many different factors can prevent women from getting medical advice or treatment for themselves. If you wanted to go to the health clinic to get or learn about a family planning method, would the following be a big problem or not: Getting permission to go to the health clinic? | 1 BIG PROBLEM |
|  |  |  | 2 NOT A BIG PROBLEM |
|  |  |  | 997 OTHER |
|  |  |  | 998 DON'T KNOW |
|  |  |  | 999 DECLINE TO ANSWER |
| 1. J_BARMONEY |  | Getting money needed for advice or treatment? | 1 BIG PROBLEM |
|  |  |  | 2 NOT A BIG PROBLEM |
|  |  |  | 997 OTHER |
|  |  |  | 998 DON'T KNOW |
|  |  |  | 999 DECLINE TO ANSWER |
| 1. J_BARDIST |  | The distance to the health facility? | 1 BIG PROBLEM |
|  |  |  | 2 NOT A BIG PROBLEM |
|  |  |  | 997 OTHER |
|  |  |  | 998 DON'T KNOW |
|  |  |  | 999 DECLINE TO ANSWER |
| 1. J_BARALONE |  | Not wanting to go alone? | 1 BIG PROBLEM |
|  |  |  | 2 NOT A BIG PROBLEM |
|  |  |  | 997 OTHER |
|  |  |  | 998 DON'T KNOW |
|  |  |  | 999 DECLINE TO ANSWER |
| 1. J_BARSTOCK |  | Fear that they would not have the family planning method I wanted in stock? | 1 BIG PROBLEM |
|  |  |  | 2 NOT A BIG PROBLEM |
|  |  |  | 997 OTHER |
|  |  |  | 998 DON'T KNOW |
|  |  |  | 999 DECLINE TO ANSWER |
| 1. J_BARSTAFF |  | 129 The fear that I would not be well received by the health staff for requesting family planning. | 1 BIG PROBLEM |
|  |  |  | 2 NOT A BIG PROBLEM |
|  |  |  | 997 OTHER |
|  |  |  | 998 DON'T KNOW |
|  |  |  | 999 DECLINE TO ANSWER |
| 1. J_BELMNURS |  | Fear that at the health center there may be a male nurse or doctor who would touch my body? | 1 BIG PROBLEM |
|  |  |  | 2 NOT A BIG PROBLEM |
|  |  |  | 997 OTHER |
|  |  |  | 998 DON'T KNOW |
|  |  |  | 999 DECLINE TO ANSWER |
| 1. J_BARPPL |  | 130 The fear that other people see me at the health center and misjudge me for requesting family planning. | 1 BIG PROBLEM |
|  |  |  | 2 NOT A BIG PROBLEM |
|  |  |  | 997 OTHER |
|  |  |  | 998 DON'T KNOW |
|  |  |  | 999 DECLINE TO ANSWER |
| **I. CONTRACEPTION USE** |  |  |  |
| 1. I_CSEVER |  | When was the last time you visited a health clinic for any type of health care for yourself? | 1 Never |
|  |  |  | 2 In the last month |
|  |  |  | 3 In the last year |
|  |  |  | 4 More than a year ago |
|  |  |  | 998 Don't know |
|  |  |  | 999 DECLINE TO ANSWER |
| 1. I_CSANTE | IF _CSEVER=2-4, 998 AND (_MISCAR=1 OR _BRTHANY=1) | Have you ever visited a health center for the purpose of receiving antenatal care? | 1 YES |
|  |  |  | 2 NO |
|  |  |  | 998 DON'T REMEMBER |
|  |  |  | 999 DECLINE TO ANSWER |
| 1. I_CSEVERFP | IF _CSEVER=2-4, 998 | Has any staff member during any of your visits at the health center ever spoken to you about family planning methods? | 1 YES |
|  |  |  | 2 NO |
|  |  |  | 998 DON'T REMEMBER |
|  |  |  | 999 DECLINE TO ANSWER |
| 1. I_CS12FP | if _CSEVER=2-3, 998 | In the last 12 months, did any staff member during any of your visits at the health center speak to you about family planning methods? | 1 YES |
|  |  |  | 2 NO |
|  |  |  | 997 OTHER |
|  |  |  | 998 DON'T REMEMBER |
|  |  |  | 999 DECLINE TO ANSWER |
| 1. I_CSINITIAT | IF _CSEVER=2-3, 998 | In the last 12 months, did you specifically go to a health clinic to learn about family planning methods? | 1 YES |
|  |  |  | 2 NO |
|  |  |  | 998 DON'T REMEMBER |
|  |  |  | 999 DECLINE TO ANSWER |
| 1. I_CSFPFP | if _CS12FP=1 OR _CSINITIAT=1 | (IF YES) Did the health center provide you with or help you start a form of family planning? | 1 No, the health center did not help me start a form of family planning |
|  |  |  | 2 IUD |
|  |  |  | 3 INJECTIBLES |
|  |  |  | 4 IMPLANTS |
|  |  |  | 5 PILL |
|  |  |  | 6 CONDOM |
|  |  |  | 7 FEMALE CONDOM |
|  |  |  | 8 EMERGENCY CONTRACEPTION |
|  |  |  | 9 LACTATION AMENAREA METHOD (LAM) |
|  |  |  | 10 OTHER TRADITIONAL METHOD |
|  |  |  | 11 ABSTINENCE |
|  |  |  | 998 DON'T KNOW |
|  |  |  | 999 DECLINE TO ANSWER |
| 1. I_CSFPTYP | IF _CSEVER=2-4, 998 | What type of health center was this? | 1 DISTRICT HOSPITAL |
|  |  |  | 2 CENTRE DE SANTÉ/CSI |
|  |  |  | 3 CASE DE SANTÉ/ACS |
|  |  |  | 4 Pharmacie |
|  |  |  | 5 Distribution à Base Communautaire/OTHER COMMUNITY HEALTH WORKER |
|  |  |  | 997 OTHER |
|  |  |  | 998 DON'T KNOW |
|  |  |  | 999 DECLINE TO ANSWER |
| 1. I_FPEVER |  | Now I would like to ask about you and your husband's use of family planning. Have you ever done something or used any method to space or delay getting pregnant? | 1 YES |
|  |  |  | 2 NO |
|  |  |  | 999 DECLINE TO ANSWER |
| 1. I_FPCURUSE | if _FPEVER=1 AND _PREG=2, 998, 999 | Are you or your husband currently doing something or using any method to space or delay getting pregnant? | 1 YES |
|  |  |  | 2 NO |
|  |  |  | 999 DECLINE TO ANSWER |
| 1. I_FPCURTYP | if _FPCURUSE=1 | Which method are you currently using? | 1 No method |
|  |  |  | 2 IUD |
|  |  |  | 3 INJECTIBLES |
|  |  |  | 4 IMPLANTS |
|  |  |  | 5 PILL |
|  |  |  | 6 CONDOM |
|  |  |  | 7 FEMALE CONDOM |
|  |  |  | 8 EMERGENCY CONTRACEPTION |
|  |  |  | 9 LACTATION AMENAREA METHOD (LAM) |
|  |  |  | 10 OTHER TRADITIONAL METHOD |
|  |  |  | 11 ABSTINENCE |
|  |  |  | 998 DON'T KNOW |
|  |  |  | 999 DECLINE TO ANSWER |
| 1. I_FPCURTYPTOT | IF _FPCURTYP=2-11 | To confirm, in total, you are currently using [TOTAL NUMBER OF CURRENT METHODS] types of family planning? | 1 CORRECT |
|  |  |  | 2 INCORRECT |
|  |  |  | 999 DECLINE TO ANSWER |
| 1. I_FPCURLAM | if _FPCURTYP=9 | (IF LAM) Do you give the baby anything to eat or drink besides breast milk? | 1 YES |
|  |  |  | 2 NO |
| 1. I_FPCURSTART | IF _FPCURTYP=2-11 | How long ago did you first start using (CURRENT METHOD)? | Number |
| 1. I_FPCURTIM | IF _FPCURTYP=2-11 | For how long have you been using (CURRENT METHOD) now without stopping? | Number |
| 1. I_FPCURLOC | if _FPCURTYP=2-8 | Where did you get the method that you are currently using? | 1 DISTRICT HOSPITAL |
|  |  |  | 2 CENTRE DE SANTÉ/CSI |
|  |  |  | 3 CASE DE SANTÉ/ACS |
|  |  |  | 4 Pharmacie |
|  |  |  | 5 Distribution à Base Communautaire/OTHER COMMUNITY HEALTH WORKER |
|  |  |  | 6 Vendeurs Ambulants |
|  |  |  | 7 Guérisseur traditionnelle |
|  |  |  | 8 FRIEND/RELATIVE |
|  |  |  | 997 OTHER (SPECIFY) |
|  |  |  | 998 DON'T KNOW |
|  |  |  | 999 DECLINE TO ANSWER |
| 1. I_FPCURLRN | IF _FPCURTYP=2-11 | Where did you learn about (CURRENT METHOD)? | 1 DISTRICT HOSPITAL |
|  |  |  | 2 CENTRE DE SANTÉ/CSI |
|  |  |  | 3 CASE DE SANTÉ/ACS |
|  |  |  | 4 Pharmacie |
|  |  |  | 5 Distribution à Base Communautaire/OTHER COMMUNITY HEALTH WORKER |
|  |  |  | 6 Vendeurs Ambulants |
|  |  |  | 7 Guérisseur traditionnelle |
|  |  |  | 8 FRIEND/RELATIVE |
|  |  |  | 997 OTHER (SPECIFY) |
|  |  |  | 998 DON'T KNOW |
|  |  |  | 999 DECLINE TO ANSWER |
| 1. I_FPCURLRNOTH | if_FPCURLRN=997 | Other_______ | Text |
| 1. I_FPPRIORA | if _FPEVER=1 | Prior to what you are doing now, have you or your husband done something or used any method to space or delay getting pregnant in the past? | 1 YES |
|  |  |  | 2 NO |
|  |  |  | 999 DECLINE TO ANSWER |
| 1. I_FPPRIORTYPA | if _FPPRIORA=1 | IF YES Which method(s) have you used? | 1 No method |
|  |  |  | 2 IUD |
|  |  |  | 3 INJECTIBLES |
|  |  |  | 4 IMPLANTS |
|  |  |  | 5 PILL |
|  |  |  | 6 CONDOM |
|  |  |  | 7 FEMALE CONDOM |
|  |  |  | 8 EMERGENCY CONTRACEPTION |
|  |  |  | 9 LACTATION AMENAREA METHOD (LAM) |
|  |  |  | 10 OTHER TRADITIONAL METHOD |
|  |  |  | 11 ABSTINENCE |
|  |  |  | 998 DON'T KNOW |
|  |  |  | 999 DECLINE TO ANSWER |
| 1. I_FPTOTUSE | IF _FPPRIORTYPA=2-11 | To confirm, in total you have used _____(TOTAL) methods to space or delay getting pregnant prior to what you are using currently. Is that correct? | 1 YES |
|  |  |  | 2 NO |
|  |  |  | 999 DECLINE TO ANSWER |
| 1. I_FPPRIORTIMA | IF _FPPRIORTYPA=2-11 | For how long did you use (METHOD)? | Number |
| 1. I_FP12TYP | IF _FPPRIORTYPA=2-11 | Have you used this method in the last 12 months? | 1 YES |
|  |  |  | 2 NO |
|  |  |  | 998 DON'T KNOW |
|  |  |  | 999 DECLINE TO ANSWER |
| 1. I_FPPRIORLRNA | IF _FPPRIORTYPA=2-11 | Where did you learn about this method? | 1 DISTRICT HOSPITAL |
|  |  |  | 2 CENTRE DE SANTÉ/CSI |
|  |  |  | 3 CASE DE SANTÉ/ACS |
|  |  |  | 4 Pharmacie |
|  |  |  | 5 Distribution à Base Communautaire/OTHER COMMUNITY HEALTH WORKER |
|  |  |  | 6 Vendeurs Ambulants |
|  |  |  | 7 Guérisseur traditionnelle |
|  |  |  | 8 FRIEND/RELATIVE |
|  |  |  | 9 group for young women |
|  |  |  | 997 OTHER (SPECIFY) |
|  |  |  | 998 DON'T KNOW |
|  |  |  | 999 DECLINE TO ANSWER |
| 1. I_FPPRIORLRNAOTH | if_FPPRIORLRNA=997 | Other_______ | Text |
| 1. I_FPPRIORLOCA | IF _FPPRIORTYPA=2-8 | Where did you get (METHOD)? | 1 DISTRICT HOSPITAL |
|  |  |  | 2 CENTRE DE SANTÉ/CSI |
|  |  |  | 3 CASE DE SANTÉ/ACS |
|  |  |  | 4 Pharmacie |
|  |  |  | 5 Distribution à Base Communautaire/OTHER COMMUNITY HEALTH WORKER |
|  |  |  | 6 Vendeurs Ambulants |
|  |  |  | 7 Guérisseur traditionnelle |
|  |  |  | 8 FRIEND/RELATIVE |
|  |  |  | 9 group for young women |
|  |  |  | 997 OTHER (SPECIFY) |
|  |  |  | 998 DON'T KNOW |
|  |  |  | 999 DECLINE TO ANSWER |
| 1. I_FPPRIORSTOPA | IF _FPPRIORTYPA=2-11 | Why did you stop using (METHOD)? | 1 NOT HAVING SEX |
|  |  |  | 2 INFREQUENT SEX |
|  |  |  | 3 CAN'T GET PREGNANT |
|  |  |  | 4 NOT MENSTRUATED SINCE LAST BIRTH |
|  |  |  | 5 BREASTFEEDING |
|  |  |  | 6 UP TO GOD/FATALISTIC |
|  |  |  | 7 RESPONDENT OPPOSED |
|  |  |  | 8 HUSBAND OPPOSED |
|  |  |  | 9 OTHERS OPPOSED |
|  |  |  | 10 RELIGIOUS PROHIBITION |
|  |  |  | 11 KNOWS NO METHOD |
|  |  |  | 12 KNOWS NO SOURCE |
|  |  |  | 13 SIDE EFFECTS/HEALTH CONCERNS |
|  |  |  | 14 LACK OF ACCESS/TOO FAR |
|  |  |  | 15 COSTS TOO MUCH |
|  |  |  | 16 PREFERRED METHOD NOT AVAILABLE |
|  |  |  | 17 NO METHOD AVAILABLE |
|  |  |  | 18 INCONVENIENT TO USE |
|  |  |  | 19 INTERFERES WITH BODY'S NORMAL PROCESSES |
|  |  |  | 20 Want children |
|  |  |  | 21 Became pregnant |
|  |  |  | 997 OTHER |
|  |  |  | 998 DON'T KNOW |
|  |  |  | 999 DECLINE TO ANSWER |
| 1. I_FPLSTSEX | if _FPEVER=1 AND _PREG=2, 998, 999 | Did you use a method of family planning the last time that you had sex with your husband? | 1 No method |
|  |  |  | 2 IUD |
|  |  |  | 3 INJECTIBLES |
|  |  |  | 4 IMPLANTS |
|  |  |  | 5 PILL |
|  |  |  | 6 CONDOM |
|  |  |  | 7 FEMALE CONDOM |
|  |  |  | 8 EMERGENCY CONTRACEPTION |
|  |  |  | 9 LACTATION AMENAREA METHOD (LAM) |
|  |  |  | 10 OTHER TRADITIONAL METHOD |
|  |  |  | 11 ABSTINENCE |
|  |  |  | 998 DON'T KNOW |
|  |  |  | 999 DECLINE TO ANSWER |
| 1. I_FPFIRST | If _FPEVER=1 | The first time you used a family planning method to delay pregnancy, how many living children did you have at that time? | Number |
| 1. I_FPHUSKN | if _FPEVER=1 | Does your husband know that you have done something or used a family planning method to space or delay pregnancy? | 1 YES |
|  |  |  | 2 NO |
|  |  |  | 997 OTHER |
|  |  |  | 998 DON'T KNOW |
|  |  |  | 999 DECLINE TO ANSWER |
| 1. I_FPHUSAPP | if _FPEVER=1 | Does your husband approve of you doing something to space or delay pregnancy? | 1 YES |
|  |  |  | 2 NO |
|  |  |  | 997 OTHER |
|  |  |  | 998 DON'T KNOW |
|  |  |  | 999 DECLINE TO ANSWER |
| 1. I_FPN3B | if _FPCURUSE=1 | (IF CURRENTLY USING) Will you continue to use (CURRENT METHOD) over the next 3 months to avoid or delay pregnancy? | 1 YES |
|  |  |  | 2 NO |
|  |  |  | 998 DON'T KNOW |
|  |  |  | 999 DECLINE TO ANSWER |
| 1. I_FPN3B2 | if _FPCURTYPTOT>1 AND _FPN3B=1 | Which of these methods will you continue to use over the next 3 months? | 1 No method |
|  |  |  | 2 IUD |
|  |  |  | 3 INJECTIBLES |
|  |  |  | 4 IMPLANTS |
|  |  |  | 5 PILL |
|  |  |  | 6 CONDOM |
|  |  |  | 7 FEMALE CONDOM |
|  |  |  | 8 EMERGENCY CONTRACEPTION |
|  |  |  | 9 LACTATION AMENAREA METHOD (LAM) |
|  |  |  | 10 OTHER TRADITIONAL METHOD |
|  |  |  | 11 ABSTINENCE |
|  |  |  | 998 DON'T KNOW |
|  |  |  | 999 DECLINE TO ANSWER |
| 1. I_FPN3A | if _FPN3B=2 OR if _FPCURUSE=2, 999 OR _PREG=2, 998, 999 | (IF NOT CURRENTLY USING) Will you use a family planning method in the next 3 months to avoid or delay pregnancy? | 1 YES |
|  |  |  | 2 NO |
|  |  |  | 998 DON'T KNOW |
|  |  |  | 999 DECLINE TO ANSWER |
| 1. I_FPNPOSTP | if _PREG=1 | After your current pregnancy is over, will you use a family planning method to space or delay pregnancy? | 1 YES |
|  |  |  | 2 NO |
|  |  |  | 998 DON'T KNOW |
|  |  |  | 999 DECLINE TO ANSWER |
| 1. I_FPN3TYP | If _FPN3A=1 OR _FPNPOSTP=1 | Which family planning method would you prefer to use? | 1 No method |
|  |  |  | 2 IUD |
|  |  |  | 3 INJECTIBLES |
|  |  |  | 4 IMPLANTS |
|  |  |  | 5 PILL |
|  |  |  | 6 CONDOM |
|  |  |  | 7 FEMALE CONDOM |
|  |  |  | 8 EMERGENCY CONTRACEPTION |
|  |  |  | 9 LACTATION AMENAREA METHOD (LAM) |
|  |  |  | 10 OTHER TRADITIONAL METHOD |
|  |  |  | 11 ABSTINENCE |
|  |  |  | 998 DON'T KNOW |
|  |  |  | 999 DECLINE TO ANSWER |
| 1. I_FPCOWIF | if _POLYTOT>1 | To the best of your knowledge, have any of the co-wives in your household ever used a family planning method? | 1 YES |
|  |  |  | 2 NO |
|  |  |  | 998 DON'T KNOW |
|  |  |  | 999 DECLINE TO ANSWER |
| 1. I_FPCOWIFTYP | If _FPCOWIF=1 | IF YES What methods have they used that you are aware of? | 1 No method |
|  |  |  | 2 IUD |
|  |  |  | 3 INJECTIBLES |
|  |  |  | 4 IMPLANTS |
|  |  |  | 5 PILL |
|  |  |  | 6 CONDOM |
|  |  |  | 7 FEMALE CONDOM |
|  |  |  | 8 EMERGENCY CONTRACEPTION |
|  |  |  | 9 LACTATION AMENAREA METHOD (LAM) |
|  |  |  | 10 OTHER TRADITIONAL METHOD |
|  |  |  | 11 ABSTINENCE |
|  |  |  | 998 DON'T KNOW |
|  |  |  | 999 DECLINE TO ANSWER |
| **K. CONTRACEPTION BELIEFS** | | | |
| 1. K_BELFPYES |  | For the next set of questions I am going to read some statements to you and I want you to tell me if you agee or disagree with them. There are no right or wrong answers, we just want to know your opinions. I believe that there is a family planning method that would help me to not get pregnant too soon after the birth of a child | 1 AGREE |
|  |  |  | 2 DISAGREE |
|  |  |  | 998 DON'T KNOW |
|  |  |  | 999 DECLINE TO ANSWER |
| 1. K_BELWAIT |  | I feel it is important to wait a healthy amount of time in between pregnancies | 1 AGREE |
|  |  |  | 2 DISAGREE |
|  |  |  | 998 DON'T KNOW |
|  |  |  | 999 DECLINE TO ANSWER |
| 1. K_BELHUSHELP |  | My husband would help me if I wanted to wait two years after giving birth to get pregnant again | 1 AGREE |
|  |  |  | 2 DISAGREE |
|  |  |  | 998 DON'T KNOW |
|  |  |  | 999 DECLINE TO ANSWER |
| 1. K_BELHUSRES |  | It is man's responsibility to make sure his wife will not get pregnant if it’s too soon since her last child was born | 1 AGREE |
|  |  |  | 2 DISAGREE |
|  |  |  | 998 DON'T KNOW |
|  |  |  | 999 DECLINE TO ANSWER |
| 1. K_BELBOTHRES |  | It is the responsibility of both the woman and her husband to healthily space pregnancy | 1 AGREE |
|  |  |  | 2 DISAGREE |
|  |  |  | 998 DON'T KNOW |
|  |  |  | 999 DECLINE TO ANSWER |
| 1. K_BELHUSDEC |  | It is the husband who should decide how many children to have. | 1 AGREE |
|  |  |  | 2 DISAGREE |
|  |  |  | 998 DON'T KNOW |
|  |  |  | 999 DECLINE TO ANSWER |
| 1. K_BELWIDEC |  | The woman has the right to decide to use a family planning method to delay pregnancy | 1 AGREE |
|  |  |  | 2 DISAGREE |
|  |  |  | 998 DON'T KNOW |
|  |  |  | 999 DECLINE TO ANSWER |
| 1. K_BELRELIGYES |  | My religion supports the healthy spacing of births | 1 AGREE |
|  |  |  | 2 DISAGREE |
|  |  |  | 998 DON'T KNOW |
|  |  |  | 999 DECLINE TO ANSWER |
| **L. SELF EFFICACY** | | | |
| 1. L_SESUGWAIT |  | For the next set of questions I am going to read some statements to you and I want you to tell me if you agee or disagree with them. There are no right or wrong answers, we just want to know your opinions. I feel confident in my ability to suggest to my husband that we wait a healthy amount of time to have another baby | 1 AGREE |
|  |  |  | 2 DISAGREE |
|  |  |  | 998 DON'T KNOW |
|  |  |  | 999 DECLINE TO ANSWER |
| 1. L_SEINITFP |  | I feel confident in my ability to suggest to my husband that we use a family planning method | 1 AGREE |
|  |  |  | 2 DISAGREE |
|  |  |  | 998 DON'T KNOW |
|  |  |  | 999 DECLINE TO ANSWER |
| 1. L_SEPERSFP |  | I feel confident in my ability to persuade my husband to allow me to use a family planning method | 1 AGREE |
|  |  |  | 2 DISAGREE |
|  |  |  | 998 DON'T KNOW |
|  |  |  | 999 DECLINE TO ANSWER |
| 1. L_SEDISAP |  | I could not continue to use a family planning method if I thought that my in-laws might find out. | 1 AGREE |
|  |  |  | 2 DISAGREE |
|  |  |  | 998 DON'T KNOW |
|  |  |  | 999 DECLINE TO ANSWER |
| 1. L_BELRELIGNO |  | I cannot use family planning because of my religion. | 1 AGREE |
|  |  |  | 2 DISAGREE |
|  |  |  | 998 DON'T KNOW |
|  |  |  | 999 DECLINE TO ANSWER |
| 1. L_SEFPGET |  | If I wanted to use a family planning method to delay getting pregnant, I feel confident in my ability to be able to get the family planning method of my choice | 1 AGREE |
|  |  |  | 2 DISAGREE |
|  |  |  | 998 DON'T KNOW |
|  |  |  | 999 DECLINE TO ANSWER |
| 1. L_SEFPUSE |  | If I wanted to use a family planning method to delay getting pregnant, I feel confident in my ability to be able to use the family planning method of my choice correctly | 1 AGREE |
|  |  |  | 2 DISAGREE |
|  |  |  | 998 DON'T KNOW |
|  |  |  | 999 DECLINE TO ANSWER |
| 1. L_SEUNEXP |  | For the next set of questions I am going to read some statements to you and I want you to tell me if you agee or disagree with them. Again, there are no right or wrong answers, we just want to know your opinions. I am confident that I could do a good job dealing with unexpected events. | 1 AGREE |
|  |  |  | 2 DISAGREE |
|  |  |  | 998 DON'T KNOW |
|  |  |  | 999 DECLINE TO ANSWER |
| 1. L_SESOLVE |  | I can solve most problems if I try hard enough | 1 AGREE |
|  |  |  | 2 DISAGREE |
|  |  |  | 998 DON'T KNOW |
|  |  |  | 999 DECLINE TO ANSWER |
| 1. L_SEFIND |  | If someone tries to keep me from getting what I want, I can find a way to get what I want. | 1 AGREE |
|  |  |  | 2 DISAGREE |
|  |  |  | 998 DON'T KNOW |
|  |  |  | 999 DECLINE TO ANSWER |
| 1. L_HOPEGD |  | I expect good things to happen to me. | 1 AGREE |
|  |  |  | 2 DISAGREE |
|  |  |  | 998 DON'T KNOW |
|  |  |  | 999 DECLINE TO ANSWER |
| 1. L_HOPEEXC |  | I am excited about my future. | 1 AGREE |
|  |  |  | 2 DISAGREE |
|  |  |  | 998 DON'T KNOW |
|  |  |  | 999 DECLINE TO ANSWER |
| 1. L_HOPEWELL |  | I trust my future will turn out well. | 1 AGREE |
|  |  |  | 2 DISAGREE |
|  |  |  | 998 DON'T KNOW |
|  |  |  | 999 DECLINE TO ANSWER |
| **M. AGENCY** | | | |
|  |  | Now, I’m going to read you some decisions that might be made in a family, and then list some family members: I will show you this picture of a ladder – imagine those at the top of the ladder have the most influence in the family over a decision and those in the bottom have no influence over that decision. For each type of decision I read to you I would like you tell me how much influence each person has for that decision. | NOT A QUESTION |
| 1. M_DECISCHILD |  | How much influence does this person have over the decision of how many children you should have? | 1 Husband |
|  |  |  | 2 Mother-in-law |
|  |  |  | 3 Wife |
|  |  |  | 4 Husband's Brother |
|  |  |  | 5 Father-in-law |
|  |  |  | 6 Co-wife |
| 1. M_DECISCHILDTOP |  | Who has the most influence in the family over the decision on how many children you should have? | Husband |
|  |  |  | Mother-in-law |
|  |  |  | Wife |
|  |  |  | Husband's Brother |
|  |  |  | Father-in-law |
|  |  |  | Co-wife |
| 1. M_DECISFP |  | Now, I’m going to read you some decisions that might be made in a family, and then list some family members: I will show you this picture of a ladder – imagine those at the top of the ladder have the most influence in the family over a decision and those in the bottom have no influence over that decision. For each type of decision I read to you I would like you tell me how much influence each person has for that decision. How much influence does this person have on the decision on whether you should use a family planning method to space births? | 1 Husband |
|  |  |  | 2 Mother-in-law |
|  |  |  | 3 Wife |
|  |  |  | 4 Husband's Brother |
|  |  |  | 5 Father-in-law |
|  |  |  | 6 Co-wife |
| 1. M_DECISFPTOP |  | Who has the most influence in the family over the decision on whether you should use a family planning method to space births? | Husband |
|  |  |  | Mother-in-law |
|  |  |  | Wife |
|  |  |  | Husband's Brother |
|  |  |  | Father-in-law |
|  |  |  | Co-wife |
| 1. M_TOPSUP | if there exists a role in _DECISCHILD that was assigned ranking of "1" | How supportive do you feel your [TOP DECISION MAKER] is in allowing you to choose when you get pregnant? | 1 SUPPORTIVE |
|  |  |  | 2 SOMEWHAT SUPPORTIVE |
|  |  |  | 3 NOT SUPPORTIVE |
|  |  |  | 998 DON'T KNOW |
|  |  |  | 999 DECLINE TO ANSWER |
| 1. M_TPNUMCONV |  | Have you ever had a conversation with your [TOP DECISION MAKER] about how many children you will have? | 1 YES |
|  |  |  | 2 NO |
|  |  |  | 998 DON'T REMEMBER |
|  |  |  | 999 DECLINE TO ANSWER |
| 1. M_TOPNUMTELL | If _TPNUMCONV=1 | (IF YES TO EVER CONVERSATION) During that conversation, did you tell your [TOP DECISION MAKER] your wishes about the number of children you will have? | 1 YES, I told him/her I want a specific number of children, a number that is 5 or fewer children |
|  |  |  | 2 YES, I told him/her I want a specific number of children, a number that is more than 5 children |
|  |  |  | 3 YES, I told him/her I want to have as many children as possible or that I do not care how many children I have |
|  |  |  | 4 NO, I did not share my wishes |
|  |  |  | 997 OTHER |
|  |  |  | 999 DECLINE TO ANSWER |
| 1. M_TOPNUMNO | if _TOPNUMTELL=4 | (IF NO) Is it because you feel your [TOP DECISION MAKER] will not take your feelings into consideration when deciding how many children you will have? | 1 YES |
|  |  |  | 2 NO |
|  |  |  | 997 OTHER (text) |
|  |  |  | 999 DECLINE TO ANSWER |
| 1. M_TOPNUMNOOTH | If _TOPNUMNO=997 | Other | Text |
| 1. M_TOPNUMWISH | if _TOPNUMTELL=1,2,3 | IF YES: Do you feel your (TOP DECISION MAKER) will take your wishes into consideration when deciding how many children you will have? | 1 YES |
|  |  |  | 2 NO |
|  |  |  | 997 OTHER (text) |
|  |  |  | 998 DON'T KNOW |
|  |  |  | 999 DECLINE TO ANSWER |
| 1. M_TOPNUMWISHOTH | If _TOPNUMWISH=997 | Other | Text |
| 1. M_TOPTMCONV | if there exists a role in _DECISFP that was assigned ranking of "1" | Have you ever had a conversation with your [TOP DECISION MAKER] about how much time you will wait between giving birth and getting pregnant again? | 1 YES |
|  |  |  | 2 NO |
|  |  |  | 998 DON'T REMEMBER |
|  |  |  | 999 DECLINE TO ANSWER |
| 1. M_TOPTM12 | if _TOPTMCONV=1 | Did you have a discussion in the past twelve months with your [TOP DECISION MAKER] about how much time you will wait between births? | 1 YES |
|  |  |  | 2 NO |
|  |  |  | 998 DON'T REMEMBER |
|  |  |  | 999 DECLINE TO ANSWER |
| 1. M_TOPTMTELL | if _TOPTMCONV=1 | (IF YES ) During that conversation, did you tell your [TOP DECISION MAKER] your wishes about how much time you will wait between births? | 1 YES, I told him/her I want to wait 2 YEARS OR MORE in between births |
|  |  |  | 2 YES, I told him/her I want to wait FEWER THAN 2 YEARS in between births |
|  |  |  | 3 YES, I told him/her I do not want to wait or do not care if I wait in between births |
|  |  |  | 4 NO, I did not share my wishes |
|  |  |  | 997 OTHER |
|  |  |  | 998 DON'T REMEMBER |
|  |  |  | 999 DECLINE TO ANSWER |
| 1. M_TOPTMNO | if _TOPTMTELL=4 | (IF NO) Is it because you feel your [TOP DECISION MAKER] will not take your feelings into consideration when deciding how much time you will wait between births? | 1 YES |
|  |  |  | 2 NO |
|  |  |  | 997 OTHER (text) |
|  |  |  | 999 DECLINE TO ANSWER |
| 1. M_TOPTMNOOTH | If _TOPTMNO=997 | Other | Text |
| 1. M_TOPTMWISH | if _TOPTMTELL=1,2,3 | IF YES: Do you feel your (TOP DECISION MAKER) will take your wishes into consideration when deciding how much time you will wait between births? | 1 YES |
|  |  |  | 2 NO |
|  |  |  | 997 OTHER (text) |
|  |  |  | 998 DON'T KNOW |
|  |  |  | 999 DECLINE TO ANSWER |
| 1. M_TOPTMWISHOTH | If _TOPTMWISH=997 | Other | Text |
| 1. M_TOPFPCONV | if there exists a role in _DECISFP that was assigned ranking of "1" | Have you ever had a conversation with your [TOP DECISION MAKER] about using a method of family planning to space births? | 1 YES |
|  |  |  | 2 NO |
|  |  |  | 998 DON'T REMEMBER |
|  |  |  | 999 DECLINE TO ANSWER |
| 1. M_TOPFP12 | If _TOPFPCONV=1 | Did you have a discussion in the past twelve months with your [TOP DECISION MAKER] about using a method of family planning to space births? | 1 YES |
|  |  |  | 2 NO |
|  |  |  | 998 DON'T REMEMBER |
|  |  |  | 999 DECLINE TO ANSWER |
| 1. M_TOPFPTELL | if _TOPFPCONV=1 | (IF YES) During that conversation, did you tell your [TOP DECISION MAKER] your wishes about using a method of family planning to space births? | 1 YES, I told him/her I want to use a family planning method |
|  |  |  | 2 YES, I told him/her I did not want to use a family planning method |
|  |  |  | 3 NO, I did not share my wishes |
|  |  |  | 997 OTHER |
|  |  |  | 998 DON'T REMEMBER |
|  |  |  | 999 DECLINE TO ANSWER |
| 1. M_TOPFPNO | if _TOPFPTELL=3 | (IF NO) Is it because you feel your [TOP DECISION MAKER] will not take your feelings into consideration when deciding your use of a method of family planning to space births? | 1 YES |
|  |  |  | 2 NO |
|  |  |  | 997 OTHER (text) |
|  |  |  | 999 DECLINE TO ANSWER |
| 1. M_TOPFPNOOTH | If _TOPFPNO=997 | Other | Text |
| 1. M_TOPFPWISH | if _TOPFPTELL=1,2 | IF YES: Do you feel your (TOP DECISION MAKER) will take your wishes into consideration when deciding your use of a method of family planning to space births? | 1 YES |
|  |  |  | 2 NO |
|  |  |  | 997 OTHER (text) |
|  |  |  | 998 DON'T KNOW |
|  |  |  | 999 DECLINE TO ANSWER |
| 1. M_TOPFPWISHOTH | If _TOPFPWISH=997 | Other | Text |
| 1. M_OTHCONV |  | Have you ever had a conversation with (other) family members or friends about things people do to delay pregnancy? | 1 YES |
|  |  |  | 2 NO |
|  |  |  | 998 DON'T REMEMBER |
|  |  |  | 999 DECLINE TO ANSWER |
| 1. M_OTHCONVW | If _OTHCONV=1 | With whom have you had a conversation about delaying pregnancy? | 1 MOTHER |
|  |  |  | 2 FRIEND |
|  |  |  | 3 SISTER |
|  |  |  | 4 MOTHER-IN-LAW |
|  |  |  | 5 CO-WIFE |
|  |  |  | 6 OTHER FEMALE RELATIVE |
|  |  |  | 7 HUSBAND |
|  |  |  | 997 OTHER |
|  |  |  | 998 DON'T REMEMBER |
|  |  |  | 999 DECLINE TO ANSWER |
| 1. M_HUSTMCONV | if HUSBAND was NOT assigned ranking of "1" (i.e. Husband is NOT top decision maker) in _DECISFP AND included in _OTHCONVW is _OTHCONVW=7 Husband | Did you have a discussion in the past twelve months with your husband about how much time you will wait between births? | 1 YES |
|  |  |  | 2 NO |
|  |  |  | 998 DON'T REMEMBER |
|  |  |  | 999 DECLINE TO ANSWER |
| 1. M_HUSTMTELL | if HUSBAND was NOT assigned ranking of "1" (i.e. Husband is NOT top decision maker) in _DECISFP AND included in _OTHCONVW is _OTHCONVW=7 Husband | (IF YES TO EVER CONVERSATION) During that conversation, did you tell him your wishes about how much time you will wait between births? | 1 YES, I told him I want to wait 2 YEARS OR MORE in between births |
|  |  |  | 2 YES, I told him I want to wait FEWER THAN 2 YEARS in between births |
|  |  |  | 3 YES, I told him I do not want to wait or do not care if I wait in between births |
|  |  |  | 4 NO, I did not share my wishes |
|  |  |  | 997 OTHER |
|  |  |  | 998 DON'T REMEMBER |
|  |  |  | 999 DECLINE TO ANSWER |
| 1. M_HUSTMNO | if _HUSTMTELL=4 | (IF NO) Is it because you feel he will not take your feelings into consideration when deciding how much time you will wait between births? | 1 YES |
|  |  |  | 2 NO |
|  |  |  | 997 OTHER (text) |
|  |  |  | 999 DECLINE TO ANSWER |
| 1. M_HUSTMNOOTH | If _HUSTMNO=997 | Other | Text |
| 1. M_HUSTMWISH | if _HUSTMTELL=1,2,3 | IF YES: Do you feel he will take your wishes into consideration when deciding how much time you will wait between births? | 1 YES |
|  |  |  | 2 NO |
|  |  |  | 997 OTHER (text) |
|  |  |  | 998 DON'T KNOW |
|  |  |  | 999 DECLINE TO ANSWER |
| 1. M_HUSTMWISHOTH | If _HUSTMWISH=997 | Other | Text |
| 1. M_HUSFPCONV | if HUSBAND was NOT assigned ranking of "1" (i.e. Husband is NOT top decision maker) in _DECISCHILD and _DECISFP AND included in _OTHCONVW is _OTHCONVW=7 Husband | (IF HUSBAND IS NOT TOP DECISION MAKER AND YES TO HUSBAND) Did you have a discussion with your husband in the past twelve months about using a method of family planning to space births? | 1 YES |
|  |  |  | 2 NO |
|  |  |  | 998 DON'T REMEMBER |
|  |  |  | 999 DECLINE TO ANSWER |
| 1. M_HUSFPTELL | if HUSBAND was NOT assigned ranking of "1" (i.e. Husband is NOT top decision maker) in _DECISCHILD and _DECISFP AND included in _OTHCONVW is _OTHCONVW=7 Husband | (IF YES TO EVER CONVERSATION) During that conversation, did you tell him your wishes about using a method of family planning to space births? | 1 YES, I told him I want to use a family planning method |
|  |  |  | 2 YES, I told him I did not want to use a family planning method |
|  |  |  | 3 NO, I did not share my wishes |
|  |  |  | 997 OTHER |
|  |  |  | 998 DON'T REMEMBER |
|  |  |  | 999 DECLINE TO ANSWER |
| 1. M_HUSFPNO | if _HUSFPTELL=3 | (IF NO) Is it because you feel he will not take your feelings into consideration when deciding your use of a method of family planning to space births? | 1 YES |
|  |  |  | 2 NO |
|  |  |  | 997 OTHER (text) |
|  |  |  | 999 DECLINE TO ANSWER |
| 1. M_HUSFPNOOTH | If _HUSFPNO=997 | Other | Text |
| 1. M_HUSFPWISH | if _HUSFPTELL=1,2 | IF YES: Do you feel he will take your wishes into consideration when deciding your use of a method of family planning to space births? | 1 YES |
|  |  |  | 2 NO |
|  |  |  | 997 OTHER (text) |
|  |  |  | 998 DON'T KNOW |
|  |  |  | 999 DECLINE TO ANSWER |
| 1. M_HUSFPWISHOTH | If _HUSFPWISH=997 | Other | Text |
| 1. M_RELQUALA |  | PLEASE READ: When two people are married, they usually share both good and bad moments. I would like to ask you some questions about your relationship with your wife. All of your responses will be kept completely confidential and will not be told to anyone. Please tell me if you agree or disagree with the following statement: [INSERT SPACE] My spouse and I talk often about problems we are facing in life. | 1 AGREE |
|  |  |  | 2 DISAGREE |
|  |  |  | 998 DON'T KNOW |
|  |  |  | 999 DECLINE TO ANSWER |
| 1. M_RELQUALD |  | My spouse blames me for things that go wrong. | 1 AGREE |
|  |  |  | 2 DISAGREE |
|  |  |  | 998 DON'T KNOW |
|  |  |  | 999 DECLINE TO ANSWER |
| 1. M_RELQUALE |  | I feel appreciated by my spouse. | 1 AGREE |
|  |  |  | 2 DISAGREE |
|  |  |  | 998 DON'T KNOW |
|  |  |  | 999 DECLINE TO ANSWER |
| 1. M_RELQUALI |  | My spouse shows love and affection for me. | 1 AGREE |
|  |  |  | 2 DISAGREE |
|  |  |  | 998 DON'T KNOW |
|  |  |  | 999 DECLINE TO ANSWER |
| 1. M_RELQUALJ |  | My spouse and I quarrel frequently | 1 AGREE |
|  |  |  | 2 DISAGREE |
|  |  |  | 998 DON'T KNOW |
|  |  |  | 999 DECLINE TO ANSWER |
| **O. VIOLENCE AGAINST WOMEN** | | | |
|  |  |  | NOT A QUESTION |
| 1. O_SXHARFEAR |  | INTRO: These next questions are about unwanted actions that may have taken place or unwanted attention you may have received, or fears you may have related to these experiences. For each question please tell me whether it never happened, happened once or twice, or happened more than twice in the last 12 months. INSERT BREAK TO SEPARATE How often are you afraid to go places because you worry about being sexually touched, harassed, or hurt by someone in these ways? Would you say: | 1 Never happened |
|  |  |  | 2 Happened once or twice |
|  |  |  | 3 Happened more than twice |
|  |  |  | 997 OTHER |
|  |  |  | 998 DON'T REMEMBER |
|  |  |  | 999 DECLINE TO ANSWER |
| 1. O_SXHARVERB |  | In the last 12 months, someone made unwanted sexual comments, jokes, or gestures towards you. Has this: | 1 Never happened |
|  |  |  | 2 Happened once or twice |
|  |  |  | 3 Happened more than twice |
|  |  |  | 997 OTHER |
|  |  |  | 998 DON'T REMEMBER |
|  |  |  | 999 DECLINE TO ANSWER |
| 1. O_SXHARPHY |  | In the last 12 months, someone touched, grabbed or pinched you in a sexual way that you did not want. Has this: | 1 Never happened |
|  |  |  | 2 Happened once or twice |
|  |  |  | 3 Happened more than twice |
|  |  |  | 997 OTHER |
|  |  |  | 998 DON'T REMEMBER |
|  |  |  | 999 DECLINE TO ANSWER |
| 1. O_SXHARLOC | if _SXHARVERB=2-5 OR _SXHARPHY=2-5 | Where has this happened? | 1 At school |
|  |  |  | 2 Inside home |
|  |  |  | 3 Compound/The immediate area around your home |
|  |  |  | 4 Your neighborhood outside compound |
|  |  |  | 5 Another neighborhood |
|  |  |  | 6 market |
|  |  |  | 7 health clinic |
|  |  |  | 8 At some other place, SPECIFY |
|  |  |  | 997 OTHER |
|  |  |  | 998 DON'T REMEMBER |
|  |  |  | 999 DECLINE TO ANSWER |
| 1. O_VIOPUSH |  | Has your husband ever done any of the following things to you: Push you, shake you, or throw something at you? | 1 YES |
|  |  |  | 2 NO |
|  |  |  | 998 DON'T KNOW |
|  |  |  | 999 DECLINE TO ANSWER |
| 1. O_VIOPUSH12 |  | Has this happened in the last 12 months? | 1 YES |
|  |  |  | 2 NO |
|  |  |  | 998 DON'T KNOW |
|  |  |  | 999 DECLINE TO ANSWER |
| 1. O_VIOSLAP |  | Slap you? | 1 YES |
|  |  |  | 2 NO |
|  |  |  | 998 DON'T KNOW |
|  |  |  | 999 DECLINE TO ANSWER |
| 1. O_VIOSLAP12 | if _VIOSLAP=1 | Has this happened in the last 12 months? | 1 YES |
|  |  |  | 2 NO |
|  |  |  | 998 DON'T KNOW |
|  |  |  | 999 DECLINE TO ANSWER |
| 1. O_VIOTWIST |  | Twist your arm or pull your hair? | 1 YES |
|  |  |  | 2 NO |
|  |  |  | 998 DON'T KNOW |
|  |  |  | 999 DECLINE TO ANSWER |
| 1. O_VIOTWIST12 | if _VIOTWIST=1 | Has this happened in the last 12 months? | 1 YES |
|  |  |  | 2 NO |
|  |  |  | 998 DON'T KNOW |
|  |  |  | 999 DECLINE TO ANSWER |
| 1. O_VIOPUNCH |  | Hit you with his fist or with something that could hurt you? | 1 YES |
|  |  |  | 2 NO |
|  |  |  | 998 DON'T KNOW |
|  |  |  | 999 DECLINE TO ANSWER |
| 1. O_VIOPUNCH12 | if _VIOPUNCH=1 | Has this happened in the last 12 months? | 1 YES |
|  |  |  | 2 NO |
|  |  |  | 998 DON'T KNOW |
|  |  |  | 999 DECLINE TO ANSWER |
| 1. O_VIOKICK |  | kick you, drag you, or beat you up? | 1 YES |
|  |  |  | 2 NO |
|  |  |  | 998 DON'T KNOW |
|  |  |  | 999 DECLINE TO ANSWER |
| 1. O_VIOKICK12 | if _VIOKICK=1 | Has this happened in the last 12 months? | 1 YES |
|  |  |  | 2 NO |
|  |  |  | 998 DON'T KNOW |
|  |  |  | 999 DECLINE TO ANSWER |
| 1. O_VIOCHOK |  | try to choke you or burn you? | 1 YES |
|  |  |  | 2 NO |
|  |  |  | 998 DON'T KNOW |
|  |  |  | 999 DECLINE TO ANSWER |
| 1. O_VIOCHOK12 | if _VIOCHOK=1 | Has this happened in the last 12 months? | 1 YES |
|  |  |  | 2 NO |
|  |  |  | 998 DON'T KNOW |
|  |  |  | 999 DECLINE TO ANSWER |
| 1. O_VIOPREG | if _BRTHANY>=1 or _PREG=1 | Has your husband ever hit, slapped, kicked, or done anything else to hurt you physically during your most recent pregnancy? | 1 YES |
|  |  |  | 2 NO |
|  |  |  | 998 DON'T KNOW |
|  |  |  | 999 DECLINE TO ANSWER |
| 1. O_VIOSX |  | Has your husband ever physically forced you to have sexual intercourse with him when you did not want to? | 1 YES |
|  |  |  | 2 NO |
|  |  |  | 998 DON'T KNOW |
|  |  |  | 999 DECLINE TO ANSWER |
| 1. O_VIOSX12 | if _VIOSX=1 | Has this happened in the last 12 months? | 1 YES |
|  |  |  | 2 NO |
|  |  |  | 998 DON'T KNOW |
|  |  |  | 999 DECLINE TO ANSWER |
| 1. O_VIOSXACT |  | Has your husband ever physically force you to perform any other sexual acts you did not want to? | 1 YES |
|  |  |  | 2 NO |
|  |  |  | 998 DON'T KNOW |
|  |  |  | 999 DECLINE TO ANSWER |
| 1. O_VIOSXACT12 | if _VIOSXACT=1 | Has this happened in the last 12 months? | 1 YES |
|  |  |  | 2 NO |
|  |  |  | 998 DON'T KNOW |
|  |  |  | 999 DECLINE TO ANSWER |
| 1. O_VIOHUM |  | Has your husband ever said or done something to humiliate you in front of others? | 1 YES |
|  |  |  | 2 NO |
|  |  |  | 998 DON'T KNOW |
|  |  |  | 999 DECLINE TO ANSWER |
| 1. O_VIOHUM12 | if _VIOHUM=1 | Has this happened in the last 12 months? | 1 YES |
|  |  |  | 2 NO |
|  |  |  | 998 DON'T KNOW |
|  |  |  | 999 DECLINE TO ANSWER |
| 1. O_VIOTHREAT |  | Has your husband ever threatened you or someone close to you with harm? | 1 YES |
|  |  |  | 2 NO |
|  |  |  | 998 DON'T KNOW |
|  |  |  | 999 DECLINE TO ANSWER |
| 1. O_VIOTHREAT12 | if _VIOTHREAT=1 | Has this happened in the last 12 months? | 1 YES |
|  |  |  | 2 NO |
|  |  |  | 998 DON'T KNOW |
|  |  |  | 999 DECLINE TO ANSWER |
| 1. O_VIOOTHER |  | Since you got married, has anyone other than your husband hit, slapped, kicked, or done anything else to hurt you physically? | 1 No one |
|  |  |  | 2 MOTHER |
|  |  |  | 3 FATHER |
|  |  |  | 4 SISTER |
|  |  |  | 5 BROTHER |
|  |  |  | 6 PREVIOUS HUSBAND/PARTNER |
|  |  |  | 7 MOTHER IN LAW |
|  |  |  | 8 FATHER IN LAW |
|  |  |  | 9 CO-WIFE |
|  |  |  | 10 OTHER FEMALE RELATIVE |
|  |  |  | 11 OTHER MALE RELATIVE |
|  |  |  | 12 FEMALE FRIEND/ACQUAINTANCE |
|  |  |  | 13 MALE FRIEND/ACQUAINTANCE |
|  |  |  | 997 OTHER |
|  |  |  | 999 DECLINE TO ANSWER |
| 1. O_GBHMCS |  | In the last 12 months, have any of the following people interfered in your ability to get health care for yourself? | 1 Never |
|  |  |  | 2 Yes, HUSBAND |
|  |  |  | 3 Yes, IN-LAWS |
|  |  |  | 4 Yes, CO-WIFE |
|  |  |  | 997 OTHER |
|  |  |  | 998 DON'T KNOW |
|  |  |  | 999 DECLINE TO ANSWER |
| 1. O_GBHMFD |  | In the last 12 months, have any of the following people stopped you from getting enough food for yourself? | 1 Never |
|  |  |  | 2 Yes, HUSBAND |
|  |  |  | 3 Yes, IN-LAWS |
|  |  |  | 4 Yes, CO-WIFE |
|  |  |  | 997 OTHER |
|  |  |  | 998 DON'T KNOW |
|  |  |  | 999 DECLINE TO ANSWER |
| 1. O_GBHMRST |  | In the last 12 months, have any of the following people stopped you from getting the rest you needed? | 1 Never |
|  |  |  | 2 Yes, HUSBAND |
|  |  |  | 3 Yes, IN-LAWS |
|  |  |  | 4 Yes, CO-WIFE |
|  |  |  | 997 OTHER |
|  |  |  | 998 DON'T KNOW |
|  |  |  | 999 DECLINE TO ANSWER |
| 1. O_GBHMCH |  | In the last 12 months, have any of the following people pressured you, made you feel badly, or treated you badly for not having a child? | 1 Never |
|  |  |  | 2 Yes, HUSBAND |
|  |  |  | 3 Yes, IN-LAWS |
|  |  |  | 4 Yes, CO-WIFE |
|  |  |  | 997 OTHER |
|  |  |  | 998 DON'T KNOW |
|  |  |  | 999 DECLINE TO ANSWER |
| 1. O_GBHMFP |  | In the last 12 months, have any of the following people ever made you feel badly or treated you badly for wanting to use a method to delay or prevent pregnancy? | 1 Never |
|  |  |  | 2 Yes, HUSBAND |
|  |  |  | 3 Yes, IN-LAWS |
|  |  |  | 4 Yes, CO-WIFE |
|  |  |  | 997 OTHER |
|  |  |  | 998 DON'T KNOW |
|  |  |  | 999 DECLINE TO ANSWER |
| 1. O_RCFORC |  | Has your husband ever tried to force or pressure you to become pregnant? | 1 YES |
|  |  |  | 2 NEVER |
|  |  |  | 998 DON'T KNOW |
|  |  |  | 999 DECLINE TO ANSWER |
| 1. O_RCFP |  | Has your husband ever taken your family planning (like pills) away from you? | 1 YES |
|  |  |  | 2 NEVER |
|  |  |  | 998 DON'T KNOW |
|  |  |  | 999 DECLINE TO ANSWER |
| 1. O_RCCLIN |  | Has your husband ever kept you from going to the clinic to get family planning | 1 YES |
|  |  |  | 2 NEVER |
|  |  |  | 998 DON'T KNOW |
|  |  |  | 999 DECLINE TO ANSWER |
| 1. O_RCLEAVE |  | Has your husband ever said he would leave you if you didn't get pregnant? | 1 YES |
|  |  |  | 2 NEVER |
|  |  |  | 998 DON'T KNOW |
|  |  |  | 999 DECLINE TO ANSWER |
| 1. O_RCVIO |  | Has your husband ever hurt you physically because you did not get pregnant? | 1 YES |
|  |  |  | 2 NEVER |
|  |  |  | 998 DON'T KNOW |
|  |  |  | 999 DECLINE TO ANSWER |
| 1. O_CHWITNES |  | Before you were married, did you ever see or hear your mother being beaten by your father or another male relative? | 1 NEVER |
|  |  |  | 2 ONLY ONCE |
|  |  |  | 3 TWO TO TEN TIMES |
|  |  |  | 4 OFTEN |
|  |  |  | 998 DON'T KNOW |
|  |  |  | 999 DECLINE TO ANSWER |
| **P. GENDER ATTITUDES BELIEFS** | | | |
| 1. P_GEMROLA |  | A woman’s most important role is to take care of the home and cook for the family. | 1 AGREE |
|  |  |  | 2 DISAGREE |
|  |  |  | 998 DON'T KNOW |
|  |  |  | 999 DECLINE TO ANSWER |
| 1. P_GEMROLB |  | A man should have the final word about decisions in the home. | 1 AGREE |
|  |  |  | 2 DISAGREE |
|  |  |  | 998 DON'T KNOW |
|  |  |  | 999 DECLINE TO ANSWER |
| 1. P_GEMROLE |  | There are times when a woman deserves to be beaten. | 1 AGREE |
|  |  |  | 2 DISAGREE |
|  |  |  | 998 DON'T KNOW |
|  |  |  | 999 DECLINE TO ANSWER |
| 1. P_GEMROLF |  | I think it is shameful when men engage in caring for children or other domestic work. | 1 AGREE |
|  |  |  | 2 DISAGREE |
|  |  |  | 998 DON'T KNOW |
|  |  |  | 999 DECLINE TO ANSWER |
| 1. P_GEMROLH |  | Giving baths to children, changing children’s clothes, and feeding children are the mother’s responsibility. | 1 AGREE |
|  |  |  | 2 DISAGREE |
|  |  |  | 998 DON'T KNOW |
|  |  |  | 999 DECLINE TO ANSWER |
| 1. P_GEMROLI |  | A woman should never question her husband’s decisions even if she disagrees with them. | 1 AGREE |
|  |  |  | 2 DISAGREE |
|  |  |  | 998 DON'T KNOW |
|  |  |  | 999 DECLINE TO ANSWER |
| 1. P_GEMROLK |  | It is natural and right that men have more power than women in the family | 1 AGREE |
|  |  |  | 2 DISAGREE |
|  |  |  | 998 DON'T KNOW |
|  |  |  | 999 DECLINE TO ANSWER |
| **N. SOCIAL NORMS** | | | |
| 1. N_MENPERSFP |  | What would be the reaction of most men in your community if their wife wanted to use a family planning method to delay or space births? They would think it is: | 1 good and help her |
|  |  |  | 2 good but do nothing |
|  |  |  | 3 bad, but do nothing |
|  |  |  | 4 bad and try to stop her |
|  |  |  | 997 OTHER |
|  |  |  | 998 DON'T KNOW |
|  |  |  | 999 DECLINE TO ANSWER |
| 1. N_COMWAIT |  | Now I am going to read a statement and I want to know if you agree or disagree with the statement. Just as before, there are no right or wrong answers. People in my village would think a young wife who waits 2 years in between having children was protecting the health of her family | 1 AGREE |
|  |  |  | 2 DISAGREE |
|  |  |  | 998 DON'T KNOW |
|  |  |  | 999 DECLINE TO ANSWER |
| 1. N_COMFP |  | People in my village would think a young wife who uses a family planning method to delay or space births was not fulfilling her duty to her family | 1 AGREE |
|  |  |  | 2 DISAGREE |
|  |  |  | 998 DON'T KNOW |
|  |  |  | 999 DECLINE TO ANSWER |
| 1. N_COMSTART |  | People in my village expect a young wife to start having children very soon after getting married, regardless of her age | 1 AGREE |
|  |  |  | 2 DISAGREE |
|  |  |  | 998 DON'T KNOW |
|  |  |  | 999 DECLINE TO ANSWER |
| 1. N_HUSREJECT |  | If a wife did not have the number of children her husband wanted, he would likely reject her. | 1 AGREE |
|  |  |  | 2 DISAGREE |
|  |  |  | 998 DON'T KNOW |
|  |  |  | 999 DECLINE TO ANSWER |
| 1. N_SNGEMA |  | People in my village think that a woman’s most important role is to take care of the home and cook for the family . | 1 AGREE |
|  |  |  | 2 DISAGREE |
|  |  |  | 998 DON'T KNOW |
|  |  |  | 999 DECLINE TO ANSWER |
| 1. N_SNGEMB |  | People in my village think that a man should have the final word about decisions in the home. | 1 AGREE |
|  |  |  | 2 DISAGREE |
|  |  |  | 998 DON'T KNOW |
|  |  |  | 999 DECLINE TO ANSWER |
| 1. N_SNGEMC |  | People in my village think that there are times when a woman deserves to be beaten. | 1 AGREE |
|  |  |  | 2 DISAGREE |
|  |  |  | 998 DON'T KNOW |
|  |  |  | 999 DECLINE TO ANSWER |
| 1. N_SNGEMD |  | People in my village think that it is shameful when men engage in caring for children or other domestic work. | 1 AGREE |
|  |  |  | 2 DISAGREE |
|  |  |  | 998 DON'T KNOW |
|  |  |  | 999 DECLINE TO ANSWER |
| 1. N_SNGEME |  | People in my village think that giving baths to children, changing children’s clothes, and feeding children are the mother’s responsibility | 1 AGREE |
|  |  |  | 2 DISAGREE |
|  |  |  | 998 DON'T KNOW |
|  |  |  | 999 DECLINE TO ANSWER |
| 1. N_SNGEMF |  | People in my village think that a woman should never question her husband’s decisions even if she disagrees with them | 1 AGREE |
|  |  |  | 2 DISAGREE |
|  |  |  | 998 DON'T KNOW |
|  |  |  | 999 DECLINE TO ANSWER |
| 1. N_SNGEMG |  | People in my village think that It is natural and right that men have more power than women in the family | 1 AGREE |
|  |  |  | 2 DISAGREE |
|  |  |  | 998 DON'T KNOW |
|  |  |  | 999 DECLINE TO ANSWER |
| 1. N_SNGEMH |  | People in my village think that if a man cooks or cleans it is shameful for his wife | 1 AGREE |
|  |  |  | 2 DISAGREE |
|  |  |  | 998 DON'T KNOW |
|  |  |  | 999 DECLINE TO ANSWER |
| 1. N_SNGEMI |  | People in my village expect that girls decide when and who to marry. | 1 AGREE |
|  |  |  | 2 DISAGREE |
|  |  |  | 998 DON'T KNOW |
|  |  |  | 999 DECLINE TO ANSWER |
| 1. N_REFSXREP |  | If a woman refuses to have sex with her husband when he wants her to, he has the right to get angry and reprimand he | 1 AGREE |
|  |  |  | 2 DISAGREE |
|  |  |  | 998 DON'T KNOW |
|  |  |  | 999 DECLINE TO ANSWER |
| 1. N_REFSXFIN |  | If a woman refuses to have sex with her husband when he wants her to, he has the right to refuse to give her money or other means of financial support | 1 AGREE |
|  |  |  | 2 DISAGREE |
|  |  |  | 998 DON'T KNOW |
|  |  |  | 999 DECLINE TO ANSWER |
| 1. N_REFSXFORC |  | If a woman refuses to have sex with her husband when he wants her to, he has the right to use force and have sex with her even if she doesn’t want to | 1 AGREE |
|  |  |  | 2 DISAGREE |
|  |  |  | 998 DON'T KNOW |
|  |  |  | 999 DECLINE TO ANSWER |
| 1. N_COMBEAT |  | People in this community expect that a husband will beat his wife | 1 AGREE |
|  |  |  | 2 DISAGREE |
|  |  |  | 998 DON'T KNOW |
|  |  |  | 999 DECLINE TO ANSWER |
| 1. N_COMVTOL |  | A woman should tolerate violence to keep her family together | 1 AGREE |
|  |  |  | 2 DISAGREE |
|  |  |  | 998 DON'T KNOW |
|  |  |  | 999 DECLINE TO ANSWER |
| 1. N_COMVPRIV |  | A man using violence against his wife is a private matter that should not be discussed outside the couple | 1 AGREE |
|  |  |  | 2 DISAGREE |
|  |  |  | 998 DON'T KNOW |
|  |  |  | 999 DECLINE TO ANSWER |
| 1. N_JUSTVOUT |  | In your opinion, is a husband justified in hitting or beating his wife in the following situations: If she goes out without telling him? | 1 YES |
|  |  |  | 2 NO |
|  |  |  | 998 DON'T KNOW |
|  |  |  | 999 DECLINE TO ANSWER |
| 1. N_JUSTVFP |  | She uses a family planning method without his permission? | 1 AGREE |
|  |  |  | 2 DISAGREE |
|  |  |  | 998 DON'T KNOW |
|  |  |  | 999 DECLINE TO ANSWER |
| 1. N_JUSTVARG |  | She argues with him? | 1 AGREE |
|  |  |  | 2 DISAGREE |
|  |  |  | 998 DON'T KNOW |
|  |  |  | 999 DECLINE TO ANSWER |
| 1. N_JUSTVSX |  | She refuses to have sex with him? | 1 AGREE |
|  |  |  | 2 DISAGREE |
|  |  |  | 998 DON'T KNOW |
|  |  |  | 999 DECLINE TO ANSWER |
| 1. N_JUSTVBRN |  | She burns his food? | 1 YES |
|  |  |  | 2 NO |
|  |  |  | 998 DON'T KNOW |
|  |  |  | 999 DECLINE TO ANSWER |
