# Supplementary material for: Spatial analysis of individual- and village-level sociodemographic characteristics associated with age at marriage among married adolescents in rural Niger
Source: BMC Public Health. 2020 May 19;20:729. doi: 10.1186/s12889-020-08759-6 (PMC7238637; doi:10.1186/s12889-020-08759-6)
Supplement: Supplementary file 2 — Additional file 2. Original survey for data collection from female participants. [file 12889_2020_8759_MOESM2_ESM.docx]

| **Household Registration and Household Survey** | | | |
| --- | --- | --- | --- |
| **Variable Name** | **Display Condition** | **Survey Question** | **Response Options** |
| **A. BACKGROUND INFORMATION/DEMOGRAPHICS** | | |  |
| 1. LANGHHR |  | Please enter language of interview | 1 Hausa |
|  |  |  | 2 Zarma |
|  |  |  | 3 French |
| 1. DISNAME |  | District Name | 1 Dosso |
|  |  |  | 2 Loga |
|  |  |  | 3 Doutchi |
| 1. VILNAME_DOSSO |  | Village Name | Select one village from the corresponding villages |
| 1. VILNAME_DOUTCHI |  |  |  |
| 1. VILNAME_LOGA |  |  |  |
| 1. VIL_LAT |  | village lattitude gps coordinate |  |
| 1. VIL_LONG |  | village longitude gps coordinate |  |
| 1. HUSNAME |  | Please enter the first name of the husband who has been selected for participation in the study. | Text |
| 1. MAGE |  | How old was [HUSBAND] at his last birthday? | Number |
| 1. MYRBRTH |  | In what year was [HUSBAND] born? | Number |
| 1. POLYTOT |  | In total, how many wives are there in this family? | Number |
| 1. CHILDTOT |  | In total, how many children does [HUSBAND] have? | Number |
| 1. WIFNAME |  | Please enter the first name of the wife who has been selected for participation in the study. | Text |
| 1. WILAST |  | Has [HUSNAME] married any of his wives after he married [WIFNAME]? | 1 Yes, he married 1 more wife after her |
|  |  |  | 2 Yes, he married 2 more wives after her |
|  |  |  | 3 Yes, he married 3 more wives after her |
|  |  |  | 4 No, she is the last wife he has married |
|  |  |  | 997 OTHER |
|  |  |  | 998 DON'T KNOW |
|  |  |  | 999 DECLINE TO ANSWER |
| 1. IWAGE |  | How old was [INDEX WIFE] at her last birthday? | Number |
| 1. IWYRBRTH |  | In what year was [INDEX WIFE] born? | Number |
| 1. IWFCH |  | How many children (boys, girls) does [INDEX WIFE] have? | Number |
| 1. PREG | if _MENARC=1 | Is [INDEX WIFE] pregnant now? | 1 YES |
|  |  |  | 2 NO |
|  |  |  | 998 DON'T KNOW |
|  |  |  | 999 DECLINE TO ANSWER |
| 1. PREGMO | if _PREG=1 | (IF YES) How many months pregnant is she? | Number |
|  | DEFAULT: assume that questions should appear for all unless condition is added; NOTE: =/ means does NOT equal |  |  |
| 1. TRIBE |  | What is your tribe or ethnic group? | 1 Hausa |
|  |  |  | 2 Zarma |
|  |  |  | 3 Tuareg |
|  |  |  | 4 Fula |
|  |  |  | 5 Kanuri |
|  |  |  | 6 Arab |
|  |  |  | 7 Tubu |
|  |  |  | 8 Gurma |
|  |  |  | 997 OTHER |
|  |  |  | 999 DECLINE TO ANSWER |
| 1. WIFAGE1, WIFAGE2, WIFAGE3, WIFAGE4 | IF _POLYTOT>1, display this question pattern for number of wives MINUS index wife (_POLYTOT-1) | INTRO: Let's talk about [HUSNAME]'S wives other than [WIFNAME]. In total there are [_POLYTOT - 1] wives besides [WIFNAME]. I am going to refer to these wives in order starting with the first wife who married [HUSNAME], but we will skip over [WIFNAME]. [SEPARATION] How old is the wife who married [HUSBAN NAME] [#].? | Number |
| 1. WIFCH1, WIFCH2, WIFCH3, WIFCH4 | IF _POLYTOT>1 | How many children (boys, girls) does wife [#] have? | Number |
| 1. MMARAGE |  | How old was [HUSBAND] when he married [INDEX WIFE]? | Number |
| 1. IWMARAGE |  | How old was [INDEX WIFE] when she married [HUSBAND]? | Number |
| 1. MSCH |  | Has [HUSBAND] ever attended modern or coranic school? | 1 YES, he attended modern school |
|  |  |  | 2 YES, he attended coranic school |
|  |  |  | 3 NO schooling, but CAN READ OR WRITE |
|  |  |  | 4 NO, he did not attend school and does not read or write |
|  |  |  | 997 OTHER |
|  |  |  | 999 DECLINE TO ANSWER |
| 1. MGRAD | if _HUSSCH=1 | IF YES What is the highest level of he school completed? | 1 INCOMPLETE PRIMARY |
|  |  |  | 2 COMPLETED PRIMARY |
|  |  |  | 3 MORE |
|  |  |  | 997 OTHER |
|  |  |  | 999 DECLINE TO ANSWER |
| 1. IWSCH |  | Has [INDEX WIFE] ever attended modern or coranic school? | 1 YES, she attended modern school |
|  |  |  | 2 YES, she attended coranic school |
|  |  |  | 3 No schooling, but CAN READ OR WRITE |
|  |  |  | 4 NO, she did not attend school and does not read or write |
|  |  |  | 997 OTHER |
|  |  |  | 999 DECLINE TO ANSWER |
| 1. IWGRAD | if _IWSCH=1 | IF YES What is the highest level of school she completed? | 1 INCOMPLETE PRIMARY |
|  |  |  | 2 COMPLETED PRIMARY |
|  |  |  | 3 MORE |
|  |  |  | 997 OTHER |
|  |  |  | 998 DON'T KNOW |
|  |  |  | 999 DECLINE TO ANSWER |
| 1. IWSCHYRS | if _IWSCH=1 or 2 | How many years to date has she attended school? | Number |
| 1. IWSCHSTOP | if _IWSCH=1 or 2 | Is she still attending school? | 1 YES |
|  |  |  | 2 NO |
|  |  |  | 997 OTHER |
|  |  |  | 998 DON'T KNOW |
|  |  |  | 999 DECLINE TO ANSWER |
| 1. IWSCHSTOPA | _IWSCHSTOP=2, 997, 999 | There are many reasons why people may have stopped attending school. I am going to ask you about some reasons people give for why some girls stop attending school. Please tell me what were the most important reasons for explaining why [INDEX WIFE] stopped attending school. | 1 She was physically or mentally challenged and unable to attend school |
|  |  |  | 2 had been very sick for 3 months or longer |
|  |  |  | 3 needed to do domestic work such as caring for younger children or sick relatives, cooking or cleaning, fetching water or wood, etc. |
|  |  |  | 4 needed to work in the field, herd animals, sell in the market, or hawk in the street |
|  |  |  | 5 needed to work for an employer |
|  |  |  | 6 there was not enough money to pay the costs of schooling |
|  |  |  | 7 the school offering the needed class was too far away |
|  |  |  | 8 because it is unsafe to travel to school |
|  |  |  | 9 because she failed examinations or had to repeat classes of schooling |
|  |  |  | 10 because of [poor] school quality |
|  |  |  | 11 because she no longer wanted to attend school |
|  |  |  | 12 because she had enough schooling |
|  |  |  | 13 because she got engaged or married |
|  |  |  | 14 because she got pregnant |
|  |  |  | 997 OTHER |
|  |  |  | 998 DON'T KNOW |
|  |  |  | 999 DECLINE TO ANSWER |
| 1. MLOCW |  | Does [HUSBAND] sleep most nights in the compound where [index wife] lives? | 1 YES |
|  |  |  | 2 NO |
|  |  |  | 997 OTHER |
|  |  |  | 999 DECLINE TO ANSWER |
| 1. MLOCP |  | Do [HUSBAND]'s parents reside in the same compound where [INDEX WIFE] lives? | 1 YES |
|  |  |  | 2 NO |
|  |  |  | 997 OTHER |
|  |  |  | 999 DECLINE TO ANSWER |
| 1. OWNWATC |  | Does any member of this household own: a) A watch? | 1 YES |
|  |  |  | 2 NO |
|  |  |  | 998 DON'T KNOW |
|  |  |  | 999 DECLINE TO ANSWER |
| 1. OWNPHON |  | b) A mobile phone? | 1 YES |
|  |  |  | 2 NO |
|  |  |  | 998 DON'T KNOW |
|  |  |  | 999 DECLINE TO ANSWER |
| 1. OWNBICY |  | c) A bicycle? | 1 YES |
|  |  |  | 2 NO |
|  |  |  | 998 DON'T KNOW |
|  |  |  | 999 DECLINE TO ANSWER |
| 1. OWNSCOO |  | d) A motor bike or motor scooter? | 1 YES |
|  |  |  | 2 NO |
|  |  |  | 998 DON'T KNOW |
|  |  |  | 999 DECLINE TO ANSWER |
| 1. OWNTRUC |  | e) A car or truck? | 1 YES |
|  |  |  | 2 NO |
|  |  |  | 998 DON'T KNOW |
|  |  |  | 999 DECLINE TO ANSWER |
| 1. OWNCART |  | f) An animal-drawn cart? | 1 YES |
|  |  |  | 2 NO |
|  |  |  | 998 DON'T KNOW |
|  |  |  | 999 DECLINE TO ANSWER |
| 1. MWORK |  | Has [HUSBAND] worked in the last 12 months? | 1 YES |
|  |  |  | 2 NO |
|  |  |  | 997 OTHER |
|  |  |  | 998 DON'T KNOW |
|  |  |  | 999 DECLINE TO ANSWER |
| 1. MOCCU | If _MWORK=1 | What is his occupation, that is, what kind of work does he mainly do? | 1 FARMING |
|  |  |  | 2 LIVESTOCK |
|  |  |  | 3 SELLING |
|  |  |  | HAWKING |
|  |  |  | 3 CONSTRUCTION |
|  |  |  | 997 OTHER |
|  |  |  | 999 DECLINE TO ANSWER |
| 1. MINCOM | If _MWORK=1 | Is he paid in cash or goods for this work or is he not paid at all? | 1 PAID |
|  |  |  | 2 NOT PAID |
|  |  |  | 997 OTHER |
|  |  |  | 998 DON'T KNOW |
|  |  |  | 999 DECLINE TO ANSWER |
| 1. MTRAV |  | In the last year, has [HUSBAND] spent a period longer than 3 months away from the village? | 1 YES |
|  |  |  | 2 NO |
|  |  |  | 997 OTHER |
|  |  |  | 998 DON'T KNOW |
|  |  |  | 999 DECLINE TO ANSWER |
| 1. MTRAVMO | if _MTRAV=1 | IF YES How long was the period that he was away? | Number |
| 1. MTRAVRE | if _MTRAV=1 | IF YES was this to work or for some other reason? | 1 WORK |
|  |  |  | 997 OTHER |
|  |  |  | 999 DECLINE TO ANSWER |
| 1. IWWORK |  | Has [INDEX WIFE] worked in the last 12 months? | 1 YES |
|  |  |  | 2 NO |
|  |  |  | 997 OTHER |
|  |  |  | 998 DON'T KNOW |
|  |  |  | 999 DECLINE TO ANSWER |
| 1. IWOCCU | if _IWWORK=1 | What is her occupation, that is, what kind of work does she mainly do? | 1 FARMING |
|  |  |  | 2 LIVESTOCK |
|  |  |  | 3 SELLING |
|  |  |  | HAWKING |
|  |  |  | 3 DOMESTIC WORK |
|  |  |  | 997 OTHER |
|  |  |  | 999 DECLINE TO ANSWER |
| 1. IWINCOM | if _IWWORK=1 | Is she paid in cash or goods for this work or is she not paid at all? | 1 PAID |
|  |  |  | 2 NOT PAID |
|  |  |  | 997 OTHER |
|  |  |  | 998 DON'T KNOW |
|  |  |  | 999 DECLINE TO ANSWER |
| 1. IWTRAV |  | In the last year, has [INDEX WIFE] spent a period longer than 3 months away from the village? | 1 YES |
|  |  |  | 2 NO |
|  |  |  | 997 OTHER |
|  |  |  | 998 DON'T KNOW |
|  |  |  | 999 DECLINE TO ANSWER |
| 1. IWTRAVMO | if _IWTRAV=1 | IF YES How long was the period that she was away? | Number |
| 1. IWTRAVRE | if _IWTRAV=1 | IF YES Was this to work or for some other reason? | 1 WORK |
|  |  |  | 2 GIVING BIRTH |
|  |  |  | 997 OTHER |
|  |  |  | 998 DON'T KNOW |
|  |  |  | 999 DECLINE TO ANSWER |
